# Centenarian controls increase variant effect sizes by an average twofold in an extreme case–extreme control analysis of Alzheimer’s disease

**DOI:** 10.1038/s41431-018-0273-5

**Published:** 2018-09-26

**Authors:** Niccolò Tesi, Sven J. van der Lee, Marc Hulsman, Iris E. Jansen, Najada Stringa, Natasja van Schoor, Hanne Meijers-Heijboer, Martijn Huisman, Philip Scheltens, Marcel J. T. Reinders, Wiesje M. van der Flier, Henne Holstege

**Affiliations:** 1 Alzheimer Center Amsterdam, Department of Neurology, Amsterdam Neuroscience, Vrije Universiteit Amsterdam, Amsterdam UMC, Amsterdam, The Netherlands; 2 Department of Clinical Genetics, Amsterdam UMC, Amsterdam, The Netherlands; 3 Delft Bioinformatics Lab, Delft University of Technology, Delft, The Netherlands; 4 Department of Complex Trait Genetics, Center for Neurogenomics and Cognitive Research, VU, Amsterdam, The Netherlands; 5 Department of Epidemiology and Biostatistics, Amsterdam UMC, Amsterdam, The Netherlands; 6 Amsterdam Public Health Research Institute, Amsterdam, The Netherlands

**Keywords:** High-throughput screening, Alzheimer's disease, Epidemiology, Geriatrics, Genetics of the nervous system

## Abstract

The detection of genetic loci associated with Alzheimer’s disease (AD) requires large numbers of cases and controls because variant effect sizes are mostly small. We hypothesized that variant effect sizes should increase when individuals who represent the extreme ends of a disease spectrum are considered, as their genomes are assumed to be maximally enriched or depleted with disease-associated genetic variants. We used 1,073 extensively phenotyped AD cases with relatively young age at onset as extreme cases (66.3 ± 7.9 years), 1,664 age-matched controls (66.0 ± 6.5 years) and 255 cognitively healthy centenarians as extreme controls (101.4 ± 1.3 years). We estimated the effect size of 29 variants that were previously associated with AD in genome-wide association studies. Comparing extreme AD cases with centenarian controls increased the variant effect size relative to published effect sizes by on average 1.90-fold (*SE* = 0.29, *p* = 9.0 × 10^−4^). The effect size increase was largest for the rare high-impact *TREM2 (R74H)* variant (6.5-fold), and significant for variants in/near *ECHDC3* (4.6-fold), *SLC24A4-RIN3* (4.5-fold), *NME8* (3.8-fold), *PLCG2* (3.3-fold), *APOE-ε2* (2.2-fold), and *APOE*-*ε4* (twofold). Comparing extreme phenotypes enabled us to replicate the AD association for 10 variants (*p* *<* 0.05) in relatively small samples. The increase in effect sizes depended mainly on using centenarians as extreme controls: the average variant effect size was not increased in a comparison of extreme AD cases and age-matched controls (0.94-fold, *p* = 6.8 × 10^−1^), suggesting that on average the tested genetic variants did not explain the extremity of the AD cases. Concluding, using centenarians as extreme controls in AD case–control studies boosts the variant effect size by on average twofold, allowing the replication of disease-association in relatively small samples.

## Introduction

Alzheimer’s disease (AD) is often characterized by a slow but progressive loss of cognitive functions, leading to loss of autonomy. [1] AD is rare at the age of 65 years, but its incidence increases exponentially to 40% at the age of 100 years. [2] It is currently the most prevalent cause of death at old age and one of the major health threats of the 21st century. [1] Better understanding of the etiological factors that determine AD is warranted as no treatment is currently available. Heritability plays an important role, as genetic factors are estimated to determine 60–80% of the risk of AD. [3] About 30% of the genetic risk is attributable to the *ε4* allele of *APOE* gene, and large collaborative efforts have identified over two dozen additional genetic loci that are associated with a slight modification of the risk of AD. [4–17] The design of these association studies relies on the comparison of very large numbers of cases with age-matched controls, such that detected associations can be attributed specifically to the disease. [18] However, given the prevalence of AD in the aging population, it is likely that a significant fraction of the controls will develop the disease at a later age. Therefore, as the AD risk for future cases likely involves the same genetic variants, using age-matched controls may quench variant association signals. This may, in part, explain the mostly small variant effect sizes associated with common variants. Also, GWAS studies mostly compare common genetic variants that are widely propagated in the population; as a consequence, these have mostly small effects on AD risk. [19] Rare genetic variants often have larger effect sizes than common variants, but as there are fewer carriers available in the population, the requirement for large sample sizes stands. [20]

The power of genetic analyses is determined by the variant frequency, the effect size of the variant, the sample size, and significance threshold set to be obtained. [21] Therefore, instead of increasing sample sizes of genetic studies to detect novel disease-associated genetic loci, an alternative strategy is to increase variant effect sizes by sampling individuals with extreme phenotypes. [20, 22, 23] For AD and other age-related diseases, extreme cases may be defined by having a relatively early age at disease onset, and having the phenotypic features characteristic for the disease, as defined by diagnostic assessment. Extreme controls are represented by individuals who reach extreme ages without the disease. [22, 24, 25] Indeed, in a case–control study of type 2 diabetes, the effect sizes for variants that were previously associated with the disease were increased when using centenarians as extreme controls. [24] The effect of using extreme phenotypes in other age-related diseases has not been studied.

Here, we explored the potential of using extreme phenotypes for genetic studies of AD by investigating the change in effect size of known AD-associated variants. Furthermore, using an age- and population-matched reference group, we investigated the contribution of each extreme phenotype.

## Methods

### Cohort description

As extreme AD cases group (denoted by *EA*), we used 1,149 AD cases from the Amsterdam Dementia Cohort (ADC). The ADC comprises patients who visit the memory clinic of the VU University Medical Center, The Netherlands. [26, 27] This cohort of AD patients is extensively characterized and comprises 503 early-onset cases (denoted by *eEA*) with an age at onset < 65 years, and 646 late-onset cases (denoted by *lEA*). Of the 503 early-onset cases, 255 had an age at onset < 60 years (*i.e.*, young early onset, denoted by *yEA*). The diagnosis of probable AD was based on the clinical criteria formulated by the National Institute of Neurological and Communicative Disorders and Stroke—Alzheimer’s Disease and Related Disorders Association (NINCDS-ADRDA) and based on National Institute of Aging–Alzheimer association (NIA-AA). [26–29] At baseline, all subjects underwent a standard clinical diagnostic assessment including neurological examination and standard blood tests. In addition, all subjects underwent magnetic resonance imaging, an electroencephalogram, and cerebrospinal fluid (CSF) [30] was analyzed for most patients. Clinical diagnosis is made in consensus-based, multidisciplinary meetings. Together, this elaborate diagnostic procedure reduces the chance of misdiagnosis. The extensive phenotyping in combination with the early disease onset generates an AD cohort that can be regarded “extreme”.

As extreme control group (denoted by *EC*), we used 268 self-reported cognitively healthy centenarians from the 100-plus Study cohort. [31] This study includes Dutch-speaking individuals who (i) can provide official evidence for being aged 100 years or older, (ii) self-report to be cognitively healthy, which is confirmed by a proxy, (iii) consent to donation of a blood sample, (iv) consent to (at least) two home visits from a researcher, and (v) consent to undergo an interview and neuropsychological test battery.

As “normal controls” (denoted by *NC*) we used 1,717 middle-aged (55–85 year-old) individuals from a representative sample of Dutch individuals from the Longitudinal Aging Study Amsterdam (LASA) cohort. [32, 33] LASA is an ongoing longitudinal study of older adults initiated in 1991, with the main objective to determine predictors and consequences of aging.

The Medical Ethics Committee of the VU University Medical Center (METC) approved the ADC cohort, the LASA study and the 100-plus Study. All participants and/or their legal guardians gave written informed consent for participation in clinical and genetic studies.

### Genotyping and imputation of 29 selected AD-associated genetic variants

We selected 29 single-nucleotide variants for which evidence for a genome-wide significant association with AD was found in previous studies (Table S1, Table S2). [4–17] Genetic variants were determined by standard genotyping or imputation methods. In brief, we genotyped all individuals using the Illumina Global Screening Array (GSAsharedCUSTOM_20018389_A2) and applied established quality control methods. [34] We used high-quality genotyping in all individuals (individual call rate > 98%, variant call rate > 98%), individuals with sex mismatches were excluded and Hardy–Weinberg equilibrium-departure was considered significant at *p* < 1 × 10^−6^. Genotypes were prepared for imputation using provided scripts (HRC-1000G-check-bim.pl). [35] This script compares variant ID, strand and allele frequencies to the haplotype reference panel (HRC v1.1, April 2016). [35] Finally, all autosomal variants were submitted to the Michigan imputation server (https://imputationserver.sph.umich.edu). [34] The server uses *SHAPEIT2* (v2.r790) to phase data and imputation to the reference panel (v1.1) was performed with *Minimac3*. [34, 36] A total of 1,149 extreme AD cases, 1,717 normal controls and 268 extreme (centenarian) controls passed quality control. Prior to analysis, we excluded individuals of non-European ancestry (*N*_*EA*_ = 67, based on 1000Genomes [37] clustering) and individuals with a family relation (*N*_*EA*_ = 9, *N*_*EC*_ = 13, *N*_*NC*_ = 53, identity-by-descent ≥ 0.3), [38] leaving 1,073 extreme AD cases (*N*_*eEA*_ = 464 and *N*_*lEA*_ = 609), 1,664 normal controls and 255 centenarian controls for the analysis.

### Statistical analysis

For each AD-associated variant, we explored the *change in effect size* (*E*) relative to reported effect sizes when (1) comparing extreme AD cases with extreme (centenarian) controls (*EA* vs *EC*); (2) comparing extreme AD cases with normal controls (*EA* vs *NC*); and (3) comparing normal AD cases with extreme (centenarian) controls (*NA* vs *EC*). To calculate variant effect sizes, we used logistic regression models correcting for population stratification (principal components 1–6). [39, 40] We calculated odds ratios relative to the Haplotype Reference Consortium (HRC) alternative allele assuming additive genetic effects, and estimated 95% confidence intervals (CIs).

We estimated the *change in effect size* relative to reported effect sizes (*E*) as follows:

1 $$E_{1 - 2}^k = \frac{{\log OR_{1 - 2}^k}}{{\log OR_l^k}}$$

where $E_{1 - 2}^k$ indicates the effect size change for variant *k* in a comparison of cohort 1 and cohort 2, *e.g*, $E_{EA - EC}^{APOE\,\varepsilon 4}$ indicates the effect size change for the *APOE ε4* variant when extreme AD cases (*EA*) are compared with cognitive healthy centenarians (*EC*). The ${\mathrm{log}}\,OR_{1 - 2}^k$denotes the effect size of variant *k* when comparing cohort 1 and cohort 2. The effect size of variant *k* reported in literature (Table S1) is denoted by ${\mathrm{log}}\,OR_l^k$.

We estimated the added value of using extreme (centenarian) controls rather than “normal age-matched controls” in a case–control analysis. For this, we wanted to compute the change in effect size when comparing non-extreme AD cases with extreme controls (*NA* vs *EC*). As we do not have direct access to “normal AD cases”, we estimated the effect size for the *NA*-*EC* comparison by summing (1) the effect size from the comparison of “normal AD cases” and “normal controls”, as reported in literature ($\log OR_l^k$), and (2) the effect size from the comparison of normal controls (*NC*) with extreme (centenarian) controls (*NC* vs *EC*), *i.e*., $\log OR_{NA - EC}^k = \log OR_l^k + \log OR_{NC - EC}^k$. The added value of using extreme controls in a case–control analysis then becomes:

2 $$E_{NA - EC}^k = \frac{{\log OR_l^k + \log OR_{NC - EC}^k}}{{\log OR_l^k}}$$

To assess whether age at disease onset had an impact on the change in effect size due to the extreme cases ($E_{EA - NC}$), we estimated the $\log OR_{eEA - NC}^k$ (early-onset extreme AD cases vs normal controls), $\log OR_{lEA - NC}^k$ (late-onset extreme AD cases vs normal controls) and the $\log OR_{yEA - NC}^k$ (younger early-onset AD cases vs normal controls), and their 95% CI. Then, we computed the probability that the effect size changes $E_{eEA - NC}^k$ and $E_{lEA - NC}^k$differed using a two-samples *z*-test (two-tailed *p* value).

### Determining significance of change in effect size

For each variant, we estimated $E_{1 - 2}^k$ and a 95% CI by sampling (*S* = 10,000) from the $\log OR_{1 - 2}^k$ and $\log OR_l^k$ based on their respective standard errors. The probability of divergence between the distributions of the $\log OR_{1 - 2}^k$and the $\log OR_l^k$ was determined using a two-sample z-test (two-tailed *p* value).

The probability of observing $E_{1 - 2}^k > 1$, *i.e.*, an increased effect size for variant *k*, is considered to be a Bernoulli variable with *p* *=* 0.5 (equal chance of having an increased/decreased effect). The number of variants that show an increase in effect ($E_{1 - 2}^k > 1$) then follows a binomial distribution.

The average change in effect size across all *K* = 29 tested variants is calculated as follows:

3 $$\bar E_{1 - 2} = \frac{1}{K}\mathop {\sum }\limits_k^K E_{1 - 2}^k$$

Confidence intervals and probability of divergence between $\bar E_{1 - 2}$ and previously reported effect sizes were estimated by sampling (*S* = 10,000, two-tailed *p* value).

Quality control of genotype data, population stratification analysis, and relatedness analyses were performed with *PLINK* (v1.90b4.6), whereas association analysis, downstream analyses, and plots were performed with *R* (v3.3.2). [41, 42]

## Results

**Fig. 1:**
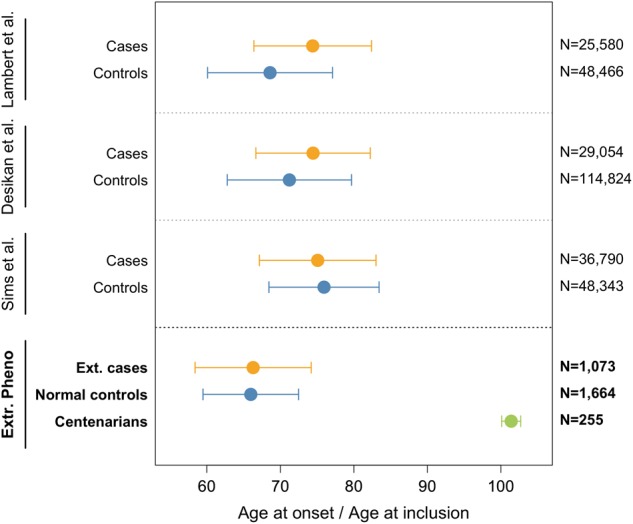
Comparison of age at disease-onset and age at inclusion for cases and controls in previously reported case-control comparisons, and in our extreme phenotypes comparison. Weighted mean and (combined) standard deviation of the age at onset for AD cases and age at inclusion for controls. As weights, we used the sample sizes of each GWA study. Note that previous case-control studies of AD included samples from multiple cohorts, sometimes overlapping across studies. References to the cohorts reported in this figure are: [7, 8, 13, 25, 26, 30]

After quality control of the genetic data, we included 1,073 extreme AD cases (with mean age at onset 66.4 ± 7.8 and 52.7% females), 1,664 normal (age-matched) controls (mean age at inclusion 66.0 ± 6.5, 53.7% females), and 255 cognitive healthy centenarians as extreme controls (mean age at inclusion 101.4 ± 1.3, 74.7% females) (Table 1). Within the extreme AD cases group, there were 464 early-onset cases (mean age at onset 59.1 ± 4.1, 54% females), and 609 late-onset cases (mean age at onset 72.1 ± 4.8, 51% females). The age at onset of the extreme AD cases was on average 8.2 years earlier compared with previous GWA studies; the age at disease onset was on average 15.4 years earlier in early-onset cases and 2.5 years earlier in late-onset cases, whereas the age at study inclusion of our centenarian controls was on average 29.5 years higher than for previously published controls (Fig. 1).

**Table 1:** Population characteristics

	Extreme AD Cases (*EA*)	Centenarian controls (*EC*)	Normal controls (*NC*)
Number of individuals	1,073	255	1,664
Females (%)	564 (52.6)	191 (74.9)	893 (53.7)
Age (SD)^a^	66.4 (7.8)	101.4 (1.3)	66.0 (6.5)
*ApoE ε4 (%)*	981 (42.7)	44 (8.6)	533 (16.0)
*ApoE ε2 (%)*	76 (3.5)	78 (15.3)	304 (9.1)

^a^Age at onset for extreme Alzheimer’s disease cases, age at study inclusion for extreme controls and normal controls; *SD,* standard deviation; *ApoE,* Apolipoprotein E allele count for *ε4* and *ε2*, respectively. Reference to the cohorts reported in this table are: [26, 27, 31, 32]

### Effect of comparing extreme cases and centenarian controls

**Fig. 2:**
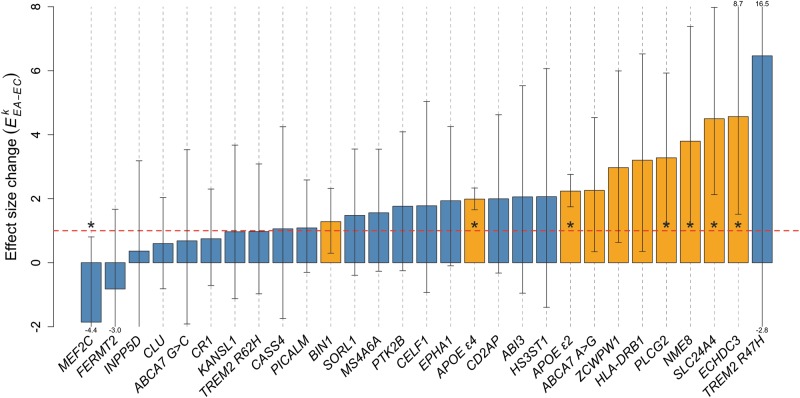
Change in variant effect-size using extreme cases and centenarian controls relative to published effect-sizes, for 29 AD associated genetic variants. Dashed red line at *E*^*k*^_*EA−EC*_ = 1 indicates same effect-size as reported in literature. Orange bars indicate nominal statistical significance for the association with AD (*p* < 0.05). Stars indicate significant changes of effect-size relative to previously reported effect-sizes (*p* < 0.05, two-sample z-test)

**Fig. 3:**
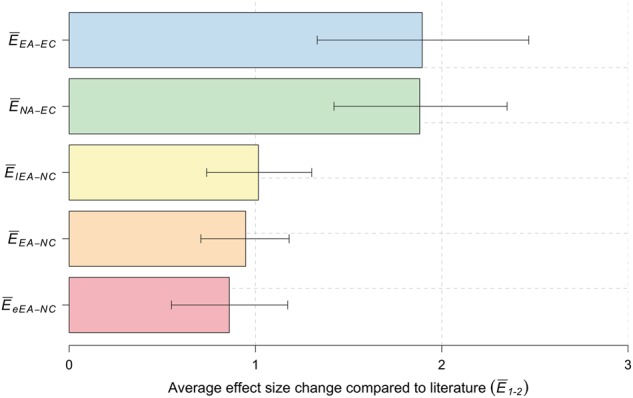
Average increase in effect-size for the different comparisons. Average increase in effect sizes for: Extreme AD cases (*N*_*EA*_ = 1,073), of which early onset cases (*N*_*eEA*_ = 464), late onset cases (*N*_*lEA*_ = 609); centenarian controls (*N*_*EC*_ = 255); normal controls (*N*_*NC*_ = 1,664). 95% confidence intervals were estimated by random sampling (*S* = 10,000)

In a genetic comparison of extreme AD cases and centenarian controls (*EA–EC* comparison) the average effect size over all 29 genetic variants was 1.90-fold increased relative to the effect sizes reported in published studies ($\bar E_{EA - EC}$ = 1.90 ± 0.29; *p* = 9.0 × 10^−4^) (Fig. 3). For 21 out of 29 variants, we observed an increased effect size ($E_{EA - EC}^k$ > 1), which is significantly more than expected by chance (*p* = 1.2 × 10^−2^) (Fig. 2 and Table 2*)*. The increase in effect size ranged from 1.06 (variant near *CASS4*) to 6.46 (variant in *TREM2 [R47H]*) and was observed both in common variants (MAF > 1%, *n* = 19) and rare variants (MAF < 1%; *TREM2 [R47H]* and *ABI3)* (Table 2). For variants near or in the genes *TREM2 (R47H)*, *SLC24A4-RIN3*, and *ECHDC3*, the increase was more than fourfold compared with previously reported effect sizes. For nine variants the effect size increase was two- to fourfold (in or near the genes *NME8*, *PLCG2*, *HLA-DRB1*, *CD2AP, ZCWPW1*, *ABCA7 [A > G], APOE [ε2]*, *HS3ST1*, and *ABI3*, in order from high to low effect size increases). For nine variants the increase was between one- and twofold (in or near genes, *APOE ε4*, *EPHA1*, *CELF1*, *PTK2B*, *MS4A6A*, *SORL1*, *BIN1*, *PICALM,* and *CASS4*) (Fig. 2). The effect sizes for six genetic variants were not increased in our extreme phenotype analysis compared with previously reported effect sizes ($E_{EA - EC}^k$ between 0 and 1): in or near *TREM2 (R62H)*, *KANSL1, CR1*, *ABCA7 (G* > *C), CLU*, and *INPP5D*. At last, the effect sizes of two variants were in the opposite direction compared to previously reported effects ($E_{EA - EC}^k$ < 0). Specifically, for the variant in *FERMT2* we found an inverted direction of effect size and a lower magnitude of effect as compared with previous studies ($E_{EA - EC}^{FERMT2}$ between 0 and − 1). For the variant near *MEF2C* we observed a larger effect size as compared with those previously published, but in the opposite direction ($E_{EA - EC}^{MEF2C}$*<* − 1).

**Table 2:** Association statistics of the 29 tested AD-associated variants

Chr	Position	Rs ID	Gene	A1	${\mathrm{log}}\,{\mathrm{OR}}_{\mathrm{l}}^{\mathrm{k}}$(SE)	${\mathrm{log}}\,{\mathrm{OR}}_{{\mathrm{EA}} - {\mathrm{EC}}}^{\mathrm{k}}$(SE)	*P* value_*EA–EC*_	${\mathrm{E}}_{{\mathrm{EA}} - {\mathrm{EC}}}^{\mathrm{k}}$ (95% CI, *p*)	AF_*EA*_	AF_*NC*_	AF_*EC*_
6	41,129,252	rs75932628	*TREM2 (R47H)*	T	0.89 (0.09)	5.75 (5.13)	2.63 × 10^−1^	6.46 (− 4.83–18.46, 3.5×10^−1^)	0.003	0.001	0.000
**10**	**11,720,308**	**rs7920721**	***ECHDC3***	**G**	**0.07 (0.01)**	**0.31 (0.10)**	**2.93** **×** **10**^**−3**^ *	**4.56 (1.55–8.93, 1.8** **×** **10**^**−2**^**)**	**0.430**	**0.389**	**0.357**
**14**	**92,926,952**	**rs10498633**	***SLC24A4-RIN3***	**T**	**−** **0.09 (0.01)**	**−** **0.42 (0.11)**	**1.30** **×** **10**^**−4**^ *	**4.50 (2.08– 7.93, 2.8** **×** **10**^**−3**^**)**	**0.206**	**0.236**	**0.292**
**7**	**37,841,534**	**rs2718058**	***NME8***	**G**	**−** **0.08 (0.01)**	**−** **0.29 (0.10)**	**3.72** **×** **10**^**−3**^ *	**3.80 (1.17**– **7.28, 3.3** **×** **10**^**−2**^**)**	**0.360**	**0.367**	**0.433**
**16**	**81,942,028**	**rs72824905**	***PLCG2***	**G**	**−** **0.39 (0.06)**	**−** **1.27 (0.40)**	**1.38** **×** **10**^**−3**^ *	**3.28 (1.26**–**5.98, 2.8×10**^**−2**^**)**	**0.008**	**0.012**	**0.025**
6	32,578,530	rs9271192	*HLA-DRB1*	A	− 0.11 (0.01)	− 0.35 (0.16)	3.06 × 10^−2^ *	3.20 (0.35–6.65, 1.3 × 10^−1^)	0.712	0.727	0.780
7	100,004,446	rs1476679	*ZCWPW1*	T	0.09 (0.01)	0.26 (0.11)	1.34 × 10^−2^ *	2.97 (0.60−6.10, 1.0 × 10^−1^)	0.703	0.674	0.649
19	1,063,443	rs4147929	*ABCA7 (A>G)*	G	− 0.14 (0.02)	− 0.32 (0.14)	2.11 × 10^−2^ *	2.26 (0.30–4.42, 2.2 × 10^−1^)	0.809	0.834	0.855
**19**	**45,412,079**	**rs7412**	***APOE (ε2)***	**T**	**−** **0.79 (0.03)**	**−** **1.76 (0.18)**	**3.16** **×** 10^−2**1**^ *	**2.24 (1.75**–**2.77, 1.4** **×** **10**^**−7**^**)**	**0.033**	**0.091**	**0.149**
4	11,711,232	rs13113697	*HS3ST1*	G	− 0.07 (0.01)	− 0.14 (0.12)	2.41 × 10^−1^	2.06 (− 1.49–6.13, 5.4×10^−1^)	0.265	0.268	0.247
17	47,297,297	rs616338	*ABI3*	C	− 0.36 (0.05)	− 0.74 (0.57)	1.93 × 10^−1^	2.06 (− 0.99–5.59, 5.2 × 10^−1^)	0.017	0.009	0.006
6	47,487,762	rs10948363	*CD2AP*	G	0.10 (0.01)	0.19 (0.11)	8.84 × 10^−2^	2.00 (− 0.34–4.60, 4.1×10^−1^)	0.284	0.272	0.245
**19**	**45,411,941**	**rs429358**	***APOE (ε4)***	**C**	**1.05 (0.03)**	**2.08 (0.17)**	**1.31** **×** **10**^**−33**^ *	**1.99 (1.65**–**2.33, 1.5** **×** **10**^**−9**^**)**	**0.429**	**0.166**	**0.082**
7	143,110,762	rs11771145	*EPHA1*	A	− 0.10 (0.01)	− 0.20 (0.10)	5.96 × 10^−2^	1.94 (− 0.09–4.29, 3.7 × 10^−1^)	0.325	0.345	0.371
11	47,557,871	rs10838725	*CELF1*	C	0.08 (0.01)	0.14 (0.11)	2.05 × 10^−1^	1.78 (− 0.95–5.11, 5.8 × 10^−1^)	0.328	0.314	0.302
8	27,195,121	rs28834970	*PTK2B*	C	0.10 (0.01)	0.18 (0.10)	8.96 × 10^−2^	1.76 (− 0.23–4.09, 4.7×10^−1^)	0.395	0.376	0.353
11	59,923,508	rs983392	*MS4A6A*	G	− 0.11 (0.01)	− 0.17 (0.10)	9.39 × 10^−2^	1.56 (− 0.20–3.61, 5.4 × 10^−1^)	0.397	0.403	0.439
11	121,435,587	rs11218343	*SORL1*	C	− 0.26 (0.03)	− 0.39 (0.25)	1.21 × 10^−1^	1.48 (− 0.39–3.51, 6.2 × 10^−1^)	0.033	0.040	0.047
2	127,892,810	rs6733839	*BIN1*	T	0.20 (0.01)	0.25 (0.10)	1.12 × 10^−2^ *	1.28 (0.31–2.29, 5.8 × 10^−1^)	0.456	0.413	0.390
11	85,867,875	rs10792832	*PICALM*	G	0.14 (0.01)	0.15 (0.10)	1.26 × 10^−1^	1.09 (− 0.30–2.56, 9.1 × 10^−1^)	0.653	0.614	0.612
20	55,018,260	rs7274581	*CASS4*	C	− 0.13 (0.02)	− 0.14 (0.18)	4.41 × 10^−1^	1.06 (−1.83–4.07, 9.7 × 10^−1^)	0.075	0.088	0.084
6	41,129,207	rs143332484	*TREM2 (R62H)*	T	0.50 (0.07)	0.48 (0.48)	3.21 × 10^−1^	0.97 (− 0.96–3.09, 9.8 × 10^−1^)	0.017	0.015	0.009
17	44,353,222	rs118172952	*KANSL1*	G	− 0.14 (0.03)	− 0.13 (0.14)	3.44 × 10^−1^	0.97 (−1.08−3.64, 9.6 × 10^−1^)	0.191	0.202	0.221
1	207,692,049	rs6656401	*CR1*	G	− 0.17 (0.01)	− 0.12 (0.12)	3.11 × 10^−1^	0.75 (− 0.75–2.21, 7.4 × 10^−1^)	0.781	0.803	0.806
19	1,061,892	rs200538373	*ABCA7 (G>C)*	C	− 0.65 (0.14)	− 0.44 (0.80)	5.81 × 10^−1^	0.68 (− 1.83–3.54, 7.9 × 10^−1^)	0.004	0.004	0.006
8	27,467,686	rs9331896	*CLU*	T	0.15 (0.01)	0.09 (0.10)	3.99 × 10^−1^	0.60 (− 0.78–2.06, 5.8 × 10^−1^)	0.361	0.400	0.378
2	234,068,476	rs35349669	*INPP5D*	T	0.08 (0.01)	0.03 (0.10)	7.83 × 10^−1^	0.36 (− 2.33–3.16, 6.2 × 10^−1^)	0.474	0.496	0.486
14	53,400,629	rs17125944	*FERMT2*	C	0.13 (0.02)	− 0.11 (0.16)	4.99 × 10^−1^	− 0.82 (− 3.46–1.60, 1.3×10^−1^)	0.104	0.105	0.114
**5**	**88,223,420**	**rs190982**	***MEF2C***	**A**	**0.08 (0.01)**	**−** **0.14 (0.10)**	**1.70** **×** **10**^**−1**^	**−** **1.86 (−** **5.01–0.77, 3.3** **×** **10**^**−2**^**)**	**0.408**	**0.406**	**0.372**
Average								1.90 ± 0.29, *p* = 9.0 x 10^−4^			

*Chr,* chromosome; *Position,* chromosomal position (GRCh37); *Rs ID,* variant ID; *Gene,* gene associated with the variant according to paper in which the variant was found; *A1,* tested allele (alternative allele according to Haplotype Reference Consortium (HRC) panel); $\log OR_l^k$*(SE),* log(odds ratio) and relative standard error for variant *k* reported by study with largest sample size; $\log OR_{EA - EC}^k$ (*SE*), log(odds ratio) and relative standard error in extreme control association; *P value*, *p* value of AD association of extreme AD cases vs centenarian controls; $E_{EA - EC}^k$ *(95% CI*, *p*), change in effect size, 95% confidence intervals and *p* value of difference when using extreme phenotypes relative to published effect sizes; AF_*EA *_, tested allele frequency in AD extreme cases; AF_*NC*_, tested allele frequency in normal controls; AF_*EC*_, tested allele frequency in centenarian controls. Bold: variants for which the *E*^*k*^_*EA-EC*_ was significantly different from published effect size; *: significant at *p* < 0.05

Overall, for seven common variants (MAF > 1%), the effect size was significantly increased relatively to the previously reported effect sizes (Table 2), in or near genes *APOE ε2* (2.2-fold, *p* = 1.4 × 10^−7^), *APOE ε4* (2.0-fold, *p* = 1.5 × 10^−9^), *SLC24A4-RIN3* (4.5-fold, *p* = 2.8 × 10^−3^), *ECHDC3* (4.6-fold, *p* = 1.8 × 10^−2^), *PLCG2* (3.3-fold *p* = 2.8 × 10^−2^), *NME8* (3.9-fold, *p* = 3.3 × 10^−2^), and *MEF2C* (−1.9-fold, *p* = 3.3 × 10^−2^). Variants with significant effect size changes were also more likely to be associated with AD in a comparison of extreme cases and centenarians. The association with AD reached nominal significance (*p* *<* 0.05) in 10 out of 21 variants with a changed effect size (Table 2). Next to *APOE ε4* ($\log OR_{EA - EC}^{APOE\,\varepsilon 4}$ = 2.1, SE = 0.17, *p* = 1.3 × 10^−33^) and *APOE ε2* ($\log OR_{EA - EC}^{APOE\,\varepsilon 2}$ = −1.8, *p* = 3.2 × 10^−21^), variants in or near these genes were significantly associated with AD: *SCL24A4-RIN3*, *PLCG2*, *ECHDC3*, *NME8*, *BIN1*, *ZCWPW1*, *ABCA7 (A* > *G)*, and *HLA-DRB1* (Table 2*)*.

### Effect of using extreme AD cases

The average effect size in a comparison of extreme AD cases with normal controls (*EA* vs *NC*) did not significantly change relative to the previously reported effect sizes ($\bar E_{EA - NC}$= 0.94 ± 0.12, *p* = 6.8 × 10^−1^) (Fig. 3). The effect size was significantly increased for *APOE ε4* variant (1.3-fold, *p* = 1.4 × 10^−5^), and nominally significant for *APOE-ε2* (1.4-fold, *p* = 1.7 × 10^−2^). For 14 individual variants, we observed an increased effect size, but this was not more than what could be expected by chance (*p* = 0.5, Figure S1 and Table S3).

We then separated AD cases into early-onset extreme AD cases (*N*_*eEA*_ = 464, age at onset < 65 years) and late-onset extreme AD cases (*N*_*lEA*_ = 609), and estimated the change in effect sizes. Unexpectedly, the average effect size in the early-onset cases was lower relative to previously published effect sizes ($\bar E_{eEA - NC}$ was 0.86 ± 0.16, *p* = 7.9 × 10^−1^), whereas for late-onset cases the effect size was similar to published effect sizes ($\bar E_{lEA - NC}$ was 1.01 ± 0.14, *p* = 4.6 × 10^−1^) (Figure S3 and Table S4). We found significant differences between the effect sizes in early-onset and late-onset AD cases ($\log OR_{eEA - NC}^k$ and $\log OR_{lEA - NC}^k$, respectively) for the variants in or near *APOE ε2* (− 0.41 vs − 0.89; *p* = 5.0 × 10^−2^), *ZCWPW1* (0.01 vs 0.24: *p* = 1.6 × 10^−2^) and *MS4A6A* (0.12 vs − 0.13; *p* = 7.9 × 10^−3^). When we extended the comparison with only the youngest early-onset AD cases (*N*_*yEA*_ = 255, age at onset < 60 years) and normal controls, the average effect size was still lower than previously published effect sizes ($\bar E_{yEA - NC}$ was 0.87 ± 0.20, *p* = 7.4 × 10^−1^) (Table S4).

### Effect of extreme controls

In a comparison of normal AD cases and extreme (centenarian) controls (*NA* vs *EC*), the effect size was on average 1.88-fold higher relative to previously reported effect sizes ($\bar E_{NA - EC}$ = 1.88 ± 0.24, *p* = 1.0 × 10^−4^) (Fig. 3). This was almost identical to the average increase in effect size when we compared the extreme cases with centenarian controls ($\bar E_{EA - EC}$ = 1.90 ± 0.29; *p* = 9.0 × 10^−4^) (Fig. 3). At the variant level, the change in effect sizes was similar in both analyses (Figure S4-A). In fact, in a comparison of normal AD cases with extreme controls, we observed an increased effect size for 24/29 variants relative to published variant effect sizes ($E_{NA - EC}^k$ > 1), which is more than expected by chance (*p* = 2.7 × 10^−4^) (Figure S2 and Table S3). As in the comparison of the extremes, we found a significant increase in effect size for variants in or near *APOE-ε2* (1.7-fold, *p* < 5 × 10^−5^), *APOE-ε4* (1.7-fold, *p* < 5 × 10^−^^5^), *NME8* (4.5-fold, *p* = *3.5* × *10*^*−3*^), *SLC24A4-RIN3* (3.9-fold, *p* = *4.5* × *10*^*−3*^) and *PLCG2* (2.9-fold, *p* = *1.9* × *10*^*−2*^). The main exception to this was the increased effect size of the rare *TREM2 (R47H)* variant (allele frequency = 0.001), which was increased more when using extreme AD cases than when using normal AD cases in a comparison with extreme controls (6.46-fold vs 3.42-fold) (Figure S4-A). For this rare variant we identified seven carriers in 1,073 extreme cases, and none in 255 centenarian controls. The effect size increase did not reach significance as CIs were large, which is according to expectations for very rare variants in small sample sizes. However, overall, the extreme controls contributed more to the effect size change than the extreme cases in a comparison of the extremes (Figure S4-B).

## Discussion

In this study, we found that the effect sizes of 29 variants previously identified in genetic case–control analyses for AD were increased in a case–control analysis of extreme phenotypes. The use of extreme AD cases and cognitively healthy centenarians as extreme controls increased effect sizes for association with AD up to sixfold, relative to previously published effect sizes. On average, the use of extreme phenotypes almost doubled the variant effect size. Although changes in effect size were different per variant, the effect size increase was driven mainly by the centenarian controls.

This profound increase enabled us to replicate the association with AD of 10 common variants in relatively small samples. In a comparison of AD cases (either normal or extreme) with centenarian controls, we observed significant effect size increases for variants in or near *PLCG2*, *NME8*, *ECHDC3*, *SLC24A4-RIN3*, *APOE-ε2*, and *APOE-ε4*. We also found a large effect size increase for the rare *TREM2* (R47H) risk variant, which did not reach significance owing to variant rareness. This suggests that the tested variants or loci might (positively or negatively) contribute to the long-term preservation of cognitive health and/or to longevity in general. *PLCG2, NME8,* and *TREM2* are implicated in immunological processes, [8, 43] whereas *SLC24A4*, *ECHDC3,* and *APOE* are involved in lipid and cholesterol metabolism (Table S5). [17, 44, 45] Both these processes were previously associated with longevity, [46, 47] such that an overlapping etiology of maintained cognitive health and maintained overall health may contribute to the observed increase in effect size. However, with the exception of the *APOE* locus, these loci were thus far not associated with longevity in GWA studies. [48–51] We speculate that the association might be dependent on the maintained cognitive health in the centenarians of the 100-plus Study cohort. [31] Alternatively, longevity studies may have been underpowered to detect the association of these loci with extreme survival. Future studies will have to establish the mechanism behind the association of these genes with preserved cognitive health. Next to *APOE*, the *HLA-DRB1* locus has been associated with both AD [13] and longevity. [48] However, its most informative variants, *rs9271192* for AD and *rs34831921* for longevity, are not in linkage disequilibrium (*r*^2^ = 0.04), suggesting that these are independent signals.

Interestingly, the variants for which the effect size did not significantly increase when using extreme cases and centenarian controls are also involved in immunity (variants in/near *TREM2*, *CR1*, *ABCA7*, *CLU*, *INPP5D*, and *MEF2C*) and lipid/cholesterol metabolism (variants in/near *ABCA7* and *CLU*) (Table S5). We speculate that variants with an increased effect size might influence changes in cognitive health during aging while variants with no increased effect size do not influence these processes.

Using extreme cases did not increase the variant effect sizes relative to published effect sizes, even though most of the extreme cases were biomarker confirmed and their mean age at onset was 8.2 years younger than the mean age at onset in other studies. [7, 8, 13] The only exception to this was the (non-significant) effect size increase for the rare *TREM2 (R47H)* risk variant, which was driven in part by using extreme AD cases. This suggests that based on the tested genetic variants, the “phenotypically extreme” cases presented in this study were not genetically more extreme than cases presented in other studies. In fact, the variant effect sizes of early-onset AD cases were on average lower than the variant effect size of late-onset AD cases, and this persists even when selecting only the youngest early-onset cases. One explanation for this observation may be that an early age at onset may be driven by rare, high-impact variants, [19] whereas the disease onset at later ages may depend to a greater extent on more common risk variants. Furthermore, we found significant differences at the variant level, between the effect sizes in early-onset and late-onset cases for common variants in/near *ZCWPW1* and *APOE ε2*, and also in —opposite directions— for the variant in *MS4A6A*. These results are a first indication that these variants may differentially influence age of disease onset, however, future experiments will have to confirm this finding.

Our main finding is that, in a genetic case–control study of extreme phenotypes, the majority of the observed increase in effect size is attributable to the extreme controls, implicating that collecting cohorts of extreme controls is profitable. We note that the centenarians used in this study were selected for their preserved cognitive health, which might have further enlarged the effect size increase for genetic variants that were previously identified for their AD association. We acknowledge that using centenarians as controls in genetic studies of AD could result in the detection of variants associated with extreme longevity, such that newly detected AD-associations need to be verified in an age-matched AD case–control setting. Nevertheless, the effect sizes for all but two variants are in the same direction as previously reported, which suggests that the tested AD variants do not have significant pleiotropic activities that counteract their AD-related survival effects. Notably, the two variants with an opposite effect, in or near *MEF2C* and *FERMT2*, also did not associate with AD in our age-matched case–control analysis. This suggests that the AD association of these variants is not consistent across studies. This is in line with results from unpublished GWASs of AD in which AD-associations of variants near the *MEF2C* and *FERMT2* genes were not replicated [52, 53] (*p* = 5.4 × 10^−3^, [52] *p* = 3.0 × 10^−4^ for *MEF2C* [53] and *p* = 1.6 × 10^−5^ for *FERMT2* [53] variant, with 5.0 × 10^−8^ being the genome-wide significance threshold). A strength of our study is that our cohorts of AD patients and controls, were not previously used in the discovery of any of the known AD-associated variants; [4–17] we thus provide independent replication in a genetically homogeneous group of individuals, as they all came from one specific population (Dutch).

Concluding, in our comparison of cases and controls with extreme phenotypes we found that on average, the effect of AD-related variants in genetic association studies almost doubled, whereas at the variant level effect sizes increased up to sixfold. The observed increment in effect size was driven by the centenarians as extreme controls, identifying centenarians as a valuable resource for genetic studies, with possible applications for other age-related diseases.

## Electronic supplementary material

Supplementary Material

## References

[1] Alzheimer’s Association. 2012 Alzheimer’s disease facts and figures. *Alzheimers Dement*. 2012;8:131–68. 10.1016/j.jalz.2012.02.001 22404854

[2] Corrada MM, Brookmeyer R, Paganini-Hill A, Berlau D, Kawas CH (2010). Dementia incidence continues to increase with age in the oldest old: The 90+study. Ann Neurol.

[3] Gatz M, Reynolds CA, Fratiglioni L (2006). Role of genes and environments for explaining Alzheimer disease. Arch Gen Psychiatry.

[4] Lambert JC, Heath S, Even G (2009). Genome-wide association study identifies variants at CLU and CR1 associated with Alzheimer’s disease. Nat Genet.

[5] Harold D, Abraham R, Hollingworth P (2009). Genome-wide association study identifies variants at CLU and PICALM associated with Alzheimer’s disease. Nat Genet.

[6] Seshadri S, Fitzpatrick AL, Ikram MA (2010). Genome-wide analysis of genetic loci associated with Alzheimer disease. JAMA.

[7] Desikan RS, Schork AJ, Wang Y (2015). Polygenic overlap between C-reactive protein, plasma lipids, and Alzheimer disease. Circulation.

[8] Sims R, van der Lee SJ, Naj AC (2017). Rare coding variants in PLCG2, ABI3, and TREM2 implicate microglial-mediated innate immunity in Alzheimer’s disease. Nat Genet.

[9] Guerreiro R, Wojtas A, Bras J (2013). TREM2 variants in Alzheimer’s disease. N Engl J Med.

[10] Jonsson T, Stefansson H, Steinberg S (2013). Variant of TREM2 associated with the risk of Alzheimer’s disease. N Engl J Med.

[11] Hollingworth P, Harold D, Sims R (2011). Common variants at ABCA7, MS4A6A/MS4A4E, EPHA1, CD33 and CD2AP are associated with Alzheimer’s disease. Nat Genet.

[12] Naj AC, Jun G, Beecham GW (2011). Common variants at MS4A4/MS4A6E, CD2AP, CD33 and EPHA1 are associated with late-onset Alzheimer’s disease. Nat Genet.

[13] Lambert JC, Ibrahim-Verbaas CA, Harold D (2013). Meta-analysis of 74,046 individuals identifies 11 new susceptibility loci for Alzheimer’s disease. Nat Genet.

[14] Jun G, Ibrahim-Verbaas CA, Vronskaya M (2016). A novel Alzheimer disease locus located near the gene encoding tau protein. Mol Psychiatry.

[15] Steinberg S, Stefansson H, Jonsson T (2015). Loss-of-function variants in ABCA7 confer risk of Alzheimer’s disease. Nat Genet.

[16] Strittmatter WJ, Saunders AM, Schmechel D (1993). Apolipoprotein E: high-avidity binding to beta-amyloid and increased frequency of type 4 allele in late-onset familial Alzheimer disease. Proc Natl Acad Sci USA.

[17] Corder EH, Saunders AM, Risch NJ (1994). Protective effect of apolipoprotein E type 2 allele for late onset Alzheimer disease. Nat Genet.

[18] Rose S, van der Laan MJ. Why Match? Investigating Matched Case-Control Study Designs with Causal Effect Estimation. *Int J Biostat.* 2009; 5:1. 10.2202/1557-4679.1127 PMC2827892 20231866

[19] Lord J, Lu AJ, Cruchaga C. Identification of rare variants in Alzheimerâ€TMs disease. *Front Genet* 2014; 5. 10.3389/fgene.2014.00369. 10.3389/fgene.2014.00369 PMC4211559 25389433

[20] Lee S, Abecasis GR, Boehnke M, Lin X (2014). Rare-variant association analysis: study designs and statistical tests. Am J Hum Genet.

[21] Hong EP, Park JW (2012). Sample size and statistical power calculation in genetic association studies. Genomics Inform.

[22] Li D, Lewinger JP, Gauderman WJ, Murcray CE, Conti D (2011). Using extreme phenotype sampling to identify the rare causal variants of quantitative traits in association studies. Genet Epidemiol.

[23] Barnett IJ, Lee S, Lin X (2013). Detecting rare variant effects using extreme phenotype sampling in sequencing association studies. Genet Epidemiol.

[24] Garagnani P, Giuliani C, Pirazzini C (2013). Centenarians as super-controls to assess the biological relevance of genetic risk factors for common age-related diseases: a proof of principle on type 2 diabetes. Aging.

[25] Peloso GM, Rader DJ, Gabriel S, Kathiresan S, Daly MJ, Neale BM (2016). Phenotypic extremes in rare variant study designs. Eur J Hum Genet.

[26] van der Flier WM, Pijnenburg YAL, Prins N (2014). Optimizing patient care and research: the Amsterdam Dementia Cohort. J Alzheimers Dis.

[27] van der Flier WM, Scheltens P (2018). Amsterdam dementia cohort: performing research to optimize care. J Alzheimers Dis.

[28] Varma AR, Snowden JS, Lloyd JJ, Talbot PR, Mann DM, Neary D (1999). Evaluation of the NINCDS-ADRDA criteria in the differentiation of Alzheimer’s disease and frontotemporal dementia. J Neurol Neurosurg Psychiatry.

[29] Blacker D, Albert MS, Bassett SS, Go RC, Harrell LE, Folstein MF (1994). Reliability and validity of NINCDS-ADRDA criteria for Alzheimer’s disease. The National Institute of Mental Health Genetics Initiative. Arch Neurol.

[30] Simonsen AH, Herukka SK, Andreasen N (2017). Recommendations for CSF AD biomarkers in the diagnostic evaluation of dementia. Alzheimers Dement.

[31] Holstege H, Beker N, Dijkstra T et al. The 100-plus Study of Dutch cognitively healthy centenarians: rationale, design and cohort description. 2018. 10.1101/295287. 10.1007/s10654-018-0451-3 PMC6290855 30362018

[32] Huisman M, Poppelaars J, van der Horst M (2011). Cohort profile: the longitudinal aging study Amsterdam. Int J Epidemiol.

[33] Hoogendijk EO, Deeg DJH, Poppelaars J (2016). The longitudinal aging study Amsterdam: cohort update 2016 and major findings. Eur J Epidemiol.

[34] Das S, Forer L, Schönherr S (2016). Next-generation genotype imputation service and methods. Nat Genet.

[35] McCarthy S, Das S, Kretzschmar W (2016). A reference panel of 64,976 haplotypes for genotype imputation. Nat Genet.

[36] O’Connell J, Gurdasani D, Delaneau O (2014). A general approach for haplotype phasing across the full spectrum of relatedness. PLoS Genet.

[37] Auton A, Brooks LD, 1000 Genomes Project Consortium (2015). A global reference for human genetic variation. Nature.

[38] Anderson CA, Pettersson FH, Clarke GM, Cardon LR, Morris AP, Zondervan KT (2010). Data quality control in genetic case–control association studies. Nat Protoc.

[39] Price AL, Zaitlen NA, Reich D, Patterson N (2010). New approaches to population stratification in genome-wide association studies. Nat Rev Genet.

[40] Price AL, Patterson NJ, Plenge RM, Weinblatt ME, Shadick NA, Reich D (2006). Principal components analysis corrects for stratification in genome-wide association studies. Nat Genet.

[41] Purcell S, Neale B, Todd-Brown K (2007). PLINK: a tool set for whole-genome association and population-based linkage analyses. Am J Hum Genet.

[42] R Core Team. R: a language and environment for statistical computing. R Foundation for Statistical Computing, Vienna, Austria.

[43] Van Cauwenberghe C, Van Broeckhoven C, Sleegers K (2016). The genetic landscape of Alzheimer disease: clinical implications and perspectives. Genet Med.

[44] Saunders AM, Strittmatter WJ, Schmechel D (1993). Association of apolipoprotein E allele epsilon 4 with late-onset familial and sporadic Alzheimer’s disease. Neurology.

[45] Kraja AT, Borecki IB, Tsai MY (2013). Genetic analysis of 16 NMR-lipoprotein fractions in humans, the GOLDN study. Lipids.

[46] Brooks-Wilson AR (2013). Genetics of healthy aging and longevity. Hum Genet.

[47] vB Hjelmborg J, Iachine I, Skytthe A (2006). Genetic influence on human lifespan and longevity. Hum Genet.

[48] Joshi PK, Pirastu N, Kentistou KA et al. Genome-wide meta-analysis associates HLA-DQA1/DRB1 and LPA and lifestyle factors with human longevity. *Nat Commun.* 2017; 8:910. 10.1038/s41467-017-00934-5 PMC5715013 29030599

[49] Ryu S, Atzmon G, Barzilai N, Raghavachari N, Suh Y (2016). Genetic landscape of APOE in human longevity revealed by high-throughput sequencing. Mech Ageing Dev.

[50] Broer L, Buchman AS, Deelen J (2015). GWAS of longevity in CHARGE consortium confirms APOE and FOXO3 candidacy. J Gerontol A Biol Sci Med Sci.

[51] Sebastiani P, Gurinovich A, Bae H (2017). Four genome-wide association studies identify new extreme longevity variantas. J Gerontol Ser A.

[52] Marioni R, Harris SE, McRae AF et al. GWAS on family history of Alzheimer’s disease. 2018;8:99. 10.1038/s41398-018-0150-6 PMC5959890 29777097

[53] Jansen I, Savage J, Watanabe K et al. Genetic meta-analysis identifies 9 novel loci and functional pathways for Alzheimers disease risk. bioRxiv. 2018. 10.1101/258533.

