# Supplementary material for: Centenarian controls increase variant effect sizes by an average twofold in an extreme case–extreme control analysis of Alzheimer’s disease
Source: Eur J Hum Genet. 2018 Sep 26;27(2):244–53. doi: 10.1038/s41431-018-0273-5 (PMC6336855; doi:10.1038/s41431-018-0273-5)
Supplement: Supplementary file 1 — Supplementary Material [file 41431_2018_273_MOESM1_ESM.pdf]

Table S1: Characteristics of the 29 genome-wide significant genetic variants associated with Alzheimer's disease.

Chr	*Pos*	*A0*	*A1*	*rs ID*	*Gene*	*AF*	*Locus discovered in*	*Effect estimate from*	$\log OR_l^k$	*Type*	*Rsq*
1	207692049	A	G	rs6656401	*CR1*	0.803	Lambert *et al.* (2009)	Lambert *et al*. (2013)	-0.17	Genotyped	-
2	127892810	C	T	rs6733839	*BIN1*	0.409	Seshadri *et al.* (2010)	Lambert *et al*. (2013)	0.20	Genotyped	-
2	234068476	C	T	rs35349669	*INPP5D*	0.512	Lambert *et al.* (2013)	Lambert *et al*. (2013)	0.08	Genotyped	-
4	11711232	T	G	rs13113697	*HS3ST1*	0.282	Desikan *et al.* (2015)	Desikan *et al*. (2015)	-0.07	Imputed	0.96
5	88223420	G	A	rs190982	*MEF2C*	0.408	Lambert *et al.* (2013)	Lambert *et al*. (2013)	0.08	Genotyped	-
6	32578530	C	A	rs9271192	*HLA-DRB1*	0.724	Lambert *et al.* (2013)	Lambert *et al*. (2013)	-0.11	Genotyped	-
6	41129207	C	T	rs143332484	*TREM2 R62H*	0.007	Sims *et al.* (2017)	Sims *et al*. (2017)	0.50	Genotyped	-
6	41129252	C	T	rs75932628	*TREM2 R47H*	0.001	Guerreiro *et al.* (2013), Jonsson *et al.* (2013)	Sims *et al*. (2017)	0.89	Imputed	0.68
6	47487762	A	G	rs10948363	*CD2AP*	0.266	Hollingworth *et al.* (2011), Naj *et al.* (2011)	Lambert *et al*. (2013)	0.10	Genotyped	-
7	37841534	A	G	rs2718058	*NME8*	0.373	Lambert *et al.* (2013)	Lambert *et al*. (2013)	-0.08	Genotyped	-
7	100004446	C	T	rs1476679	*ZCWPW1*	0.713	Lambert *et al.* (2013)	Lambert *et al*. (2013)	0.09	Genotyped	-
7	143110762	G	A	rs11771145	*EPHA1*	0.338	Hollingworth *et al.* (2011), Naj *et al.* (2011)	Lambert *et al*. (2013)	-0.10	Genotyped	-
8	27195121	T	C	rs28834970	*PTK2B*	0.366	Lambert *et al.* (2013)	Lambert *et al*. (2013)	0.10	Genotyped	-
8	27467686	C	T	rs9331896	*CLU*	0.379	Harold *et al.* (2009), Lambert *et al.* (2009)	Lambert *et al*. (2013)	0.15	Genotyped	-
10	11720308	A	G	rs7920721	*ECHDC3*	0.387	Desikan *et al.* (2015)	Desikan *et al*. (2015)	0.07	Imputed	0.98
11	47557871	T	C	rs10838725	*CELF1*	0.316	Lambert *et al.* (2013)	Lambert *et al*. (2013)	0.08	Genotyped	-
11	59923508	A	G	rs983392	*MS4A6A*	0.403	Hollingworth *et al.* (2011), Naj *et al.* (2011)	Lambert *et al*. (2013)	-0.10	Genotyped	-
11	85867875	A	G	rs10792832	*PICALM*	0.642	Harold *et al.* (2009)	Lambert *et al*. (2013)	0.14	Genotyped	-
11	121435587	T	C	rs11218343	*SORL1*	0.039	Lambert *et al.* (2013)	Lambert *et al.* (2013)	-0.26	Genotyped	-
14	53400629	T	C	rs17125944	*FERMT2*	0.092	Lambert *et al.* (2013)	Lambert *et al.* (2013)	0.13	Genotyped	-
14	92926952	G	T	rs10498633	*SLC24A4-RIN3*	0.217	Lambert *et al.* (2013)	Lambert *et al.* (2013)	-0.09	Genotyped	-
16	81942028	C	G	rs72824905	*PLCG2*	0.015	Sims *et al.* (2017)	Sims *et al.* (2017)	-0.39	Imputed	0.88
17	44353222	T	G	rs2732703	*KANSL1*	0.127	Jun *et al.* (2016)	Lambert *et al.* (2013)	-0.14	Imputed	0.81
17	47297297	T	C	rs616338	*ABI3*	0.006	Sims *et al.* (2017)	Sims *et al.* (2017)	-0.36	Imputed	0.71
19	1061892	G	C	rs200538373	*ABCA7 G>C*	0.001	Steinberg *et al.* (2015)	Steinberg *et al.* (2015)	-0.65	Imputed	0.76
19	1063443	A	G	rs4147929	*ABCA7 A>G*	0.810	Hollingworth *et al.* (2011), Naj *et al.* (2011)	Lambert *et al.* (2013)	-0.14	Imputed	0.99
19	45411941	T	C	rs429358	*ApoE Ɛ4*	0.148	Saunders *et al.* (1993), Strittmatter *et al.* (1993)	Sims *et al.* (2017)	1.05	Imputed	0.99
19	45412079	C	T	rs7412	*ApoE Ɛ2*	0.072	Corder *et al.* (1994)	Sims *et al.* (2017)	-0.79	Genotyped	-
20	55018260	T	C	rs7274581	*CASS4*	0.083	Lambert *et al.* (2013)	Lambert *et al.* (2013)	-0.13	Genotyped	-

Abbreviations: *Chr*, chromosome; *Pos*, chromosomal position (GRCh37, hg19); *A0*, reference allele in HRC reference panel; *A1*, tested allele (alternative allele in HRC reference panel); *rs ID*, variant ID; *Gene*, gene associated with the variant; *AF,* frequency of A1 in HRC reference panel; $\log OR_i^k$, published log(odds ratio) and relative standard errors, *i.e* the first or largest case-control study that reported the variant association with AD; *Type*, whether the variant was genotyped or imputed in our datasets; *Rsq*, imputation quality for imputed variants. References to the papers reported in this table are: [4–17]**.**

Table S2: Human Genome Variation Society (HGVS) codes for the tested variants

Genomic position	Rs ID	Ref HRC	Alt HRC	HGVS nomenclature
1:207692049	rs6656401	A	G	NC_000001.10:g.207692049A>G
2:127892810	rs6733839	C	T	NC_000002.11:g.127892810C>T
2:234068476	rs35349669	C	T	NC_000002.11:g.234068476C>T
4:11711232	rs13113697	T	G	NC_000004.11:g.11711232T>G
5:88223420	rs190982	G	A	NC_000005.9:g.88223420G>A
6:32578530	rs9271192	C	A	NT_113891.2:g.4029235A>C
6:41129207	rs143332484	C	T	NC_000006.11:g.41129207C>T
6:41129252	rs75932628	C	T	NC_000006.11:g.41129252C>T
6:47487762	rs10948363	A	G	NC_000006.11:g.47487762A>G
7:37841534	rs2718058	A	G	NC_000007.13:g.37841534A>G
7:100004446	rs1476679	C	T	NC_000007.13:g.100004446C>T
7:143110762	rs11771145	G	A	NC_000007.13:g.143110762G>A
8:27195121	rs28834970	T	C	NC_000008.10:g.27195121T>C
8:27467686	rs9331896	C	T	NC_000008.10:g.27467686C>T
10:11720308	rs7920721	A	G	NC_000010.10:g.11720308A>G
11:47557871	rs10838725	T	C	NC_000011.9:g.47557871T>C
11:59923508	rs983392	A	G	NC_000011.9:g.59923508A>G
11:85867875	rs10792832	A	G	NC_000011.9:g.85867875A>G
11:121435587	rs11218343	T	C	NC_000011.9:g.121435587T>C
14:53400629	rs17125944	T	C	NC_000014.8:g.53400629T>C
14:92926952	rs10498633	G	T	NC_000014.8:g.92926952G>T
16:81942028	rs72824905	C	G	NC_000016.9:g.81942028C>G
17:44353222	rs2732703	T	G	NC_000017.10:g.44353222T>G
17:47297297	rs616338	T	C	NC_000017.10:g.47297297T>C
19:1061892	rs200538373	G	C	NC_000019.9:g.1061892G>C
19:1063443	rs4147929	A	G	NC_000019.9:g.1063443A>G
19:45411941	rs429358	T	C	NC_000019.9:g.45411941T>C
19:45412079	rs7412	C	T	NC_000019.9:g.45412079C>T
20:55018260	rs7274581	T	C	NC_000020.10:g.55018260T>C

Abbreviations: Genomic position, chromosome and position an GRCh37 (hq19); Rs ID, variant identifier; Ref HRC, reference allele in Human Reference Consortium (HRC) panel; Alt HRC, alternative allele in HRC panel; HGVS nomenclature, official nomenclature according to Human Genome Variation Society (HGVS) and link to dbSNP database.

Table S3: Comparison between using extreme AD cases compared to using extreme controls in association analyses

			Extreme AD cases *vs.* Normal Controls *(EA vs. NC)*			Normal AD cases *vs.* Extreme Controls *(NA vs. EC)*		
Rs ID	***Gene***	$\log OR_l^k$ ***(SE)***	$\log OR_{EA-NC}^k$ ***(SE)***	***P-value$_{EA-NC}$***	E_{EA-NC}^k ***(95% CI, p)***	$\log OR_{NA-EC}^k$ ***(SE)***	***P-value$_{NC-EC}$#***	E_{NA-EC}^k ***(95% CI, p)***
rs75932628	*TREM2 (R47H)*	0.89 (0.09)	1.56 (0.66)	$1.86x10^{-2}$ *	1.76 (0.28 - 3.34, $3.2x10^{-1}$)	3.04 (2.75)	$5.11x10^{-1}$	3.42 (-3.85 - 10.92, $5.0x10^{-1}$)
rs7920721	*ECHDC3*	0.07 (0.01)	0.17 (0.05)	$2.61x10^{-3}$ *	2.54 (0.84 - 4.96, $7.8x10^{-2}$)	0.21 (0.08)	$1.51x10^{-1}$	3.14 (0.26 - 6.60, $1.5x10^{-1}$)
rs10498633	*SLC24A4-RIN3*	-0.09 (0.01)	-0.18 (0.07)	$8.95x10^{-3}$ *	1.85 (0.46 - 3.79, $2.4x10^{-1}$)	**-0.38 (0.46)**	**$8.59x10^{-3}$ ***	**3.93 (1.67 - 6.81, $9.2x10^{-3}$)**
rs2718058	*NME8*	-0.08 (0.01)	-0.03 (0.06)	$6.45x10^{-1}$	0.35 (-1.16 - 1.91, $3.8x10^{-1}$)	**-0.34 (0.08)**	**$5.99x10^{-3}$ ***	**4.46 (1.87 - 7.90, $7.6x10^{-3}$)**
rs72824905	*PLCG2*	-0.39 (0.06)	-0.47 (0.30)	$1.23x10^{-1}$	1.21 (-0.33 - 3.00, $7.7x10^{-1}$)	**-1.13 (0.29)**	**$3.47x10^{-2}$ ***	**2.89 (1.14 - 5.07, $3.4x10^{-2}$)**
rs9271192	*HLA-DRB1*	-0.11 (0.01)	-0.13 (0.09)	$1.40x10^{-1}$	1.19 (-0.42 - 3.01, $8.0x10^{-1}$)	-0.34 (0.13)	$1.33x10^{-1}$	3.18 (0.38 - 6.49, $1.4x10^{-1}$)
rs1476679	*ZCWPW1*	0.09 (0.01)	0.14 (0.06)	$1.94x10^{-2}$ *	1.59 (0.23 - 3.25, $3.9x10^{-1}$)	0.20 (0.08)	$2.45x10^{-1}$	2.30 (0.10 - 4.86, $2.4x10^{-1}$)
rs4147929	*ABCA7 (A>G)*	-0.14 (0.02)	-0.17 (0.07)	$2.13x10^{-2}$ *	1.18 (0.18 - 2.35, $7.4x10^{-1}$)	-0.31 (0.11)	$2.20x10^{-1}$	2.16 (0.27 - 4.13, $2.2x10^{-1}$)
rs7412	*APOE (ε2)*	-0.79 (0.03)	**-1.09 (0.14)**	**$2.36x10^{-15}$ ***	**1.38 (1.02 - 1.76, $3.7x10^{-2}$)**	**-1.37 (0.12)**	**$3.46x10^{-5}$ ***	**1.75 (1.39 - 2.11, $1.5x10^{-4}$)**
rs13113697	*HS3ST1*	-0.07 (0.01)	0.00 (0.06)	$9.70x10^{-1}$	0.04 (-1.97 - 2.04, $3.1x10^{-1}$)	-0.18 (0.09)	$3.12x10^{-1}$	2.70 (-0.60 - 6.53, $3.0x10^{-1}$)
rs616338	*ABI3*	-0.36 (0.05)	-0.70 (0.28)	$1.23x10^{-2}$ *	1.92 (0.41 - 3.79, $2.4x10^{-1}$)	-0.37 (0.47)	$9.87x10^{-1}$	1.03 (-2.10 - 4.19, $9.7x10^{-1}$)
rs10948363	*CD2AP*	0.10 (0.01)	0.07 (0.06)	$2.79x10^{-1}$	0.70 (-0.54 - 2.11, $6.5x10^{-1}$)	0.22 (0.09)	$2.78x10^{-1}$	2.28 (-0.01 - 4.83, $2.7x10^{-1}$)
rs429358	*APOE (ε4)*	1.05 (0.03)	**1.36 (0.07)**	**$1.19x10^{-84}$ ***	**1.30 (1.16 - 1.45, $1.4x10^{-5}$)**	**1.84 (0.14)**	**$4.22x10^{-6}$ ***	**1.75 (1.43 - 2.07, $1.8x10^{-5}$)**
rs11771145	*EPHA1*	-0.10 (0.01)	-0.09 (0.06)	$1.12x10^{-1}$	0.93 (-0.21 - 2.18, $8.9x10^{-1}$)	-0.23 (0.08)	$2.06x10^{-1}$	2.24 (0.26 - 4.32, $2.1x10^{-1}$)
rs10838725	*CELF1*	0.08 (0.01)	0.07 (0.06)	$2.49x10^{-1}$	0.87 (-0.61 - 2.58, $8.5x10^{-1}$)	0.13 (0.09)	$5.92x10^{-1}$	1.71 (-1.01 - 4.60, $5.9x10^{-1}$)
rs28834970	*PTK2B*	0.10 (0.01)	0.08 (0.06)	$1.44x10^{-1}$	0.84 (-0.27 - 2.08, $7.9x10^{-1}$)	0.20 (0.08)	$3.04x10^{-1}$	2.03 (0.08 - 4.12, $3.0x10^{-1}$)
rs983392	*MS4A6A*	-0.11 (0.01)	-0.02 (0.06)	$7.08x10^{-1}$	0.20 (-0.89 - 1.27, $1.4x10^{-1}$)	-0.26 (0.08)	$1.16x10^{-1}$	2.40 (0.66 - 4.33, $1.1x10^{-1}$)
rs11218343	*SORL1*	-0.26 (0.03)	-0.21 (0.15)	$1.55x10^{-1}$	0.82 (-0.30 - 2.03, $7.3x10^{-1}$)	-0.42 (0.19)	$4.71x10^{-1}$	1.63 (-0.09 - 3.42, $4.6x10^{-1}$)
rs6733839	*BIN1*	0.20 (0.01)	0.17 (0.06)	$3.04x10^{-3}$ *	0.84 (0.27 - 1.42, $5.8x10^{-1}$)	0.29 (0.08)	$3.22x10^{-1}$	1.49 (0.55 - 2.48, $3.1x10^{-1}$)
rs10792832	*PICALM*	0.14 (0.01)	0.17 (0.06)	$3.25x10^{-3}$ *	1.20 (0.40 - 2.07, $6.3x10^{-1}$)	0.15 (0.08)	$8.81x10^{-1}$	1.10 (-0.28 - 2.49, $8.8x10^{-1}$)
rs7274581	*CASS4*	-0.13 (0.02)	-0.16 (0.10)	$1.08x10^{-1}$	1.24 (-0.24 - 3.10, $7.5x10^{-1}$)	-0.09 (0.14)	$8.18x10^{-1}$	0.70 (-1.96 - 3.33, $8.2x10^{-1}$)
rs143332484	*TREM2 (R62H)*	0.50 (0.07)	0.09 (0.22)	$6.76x10^{-1}$	0.19 (-0.71 - 1.10, $7.8x10^{-2}$)	0.95 (0.39)	$3.40x10^{-1}$	1.92 (0.05 - 3.95, $3.4x10^{-1}$)
rs118172952	*KANSL1*	-0.14 (0.03)	-0.08 (0.08)	$3.32x10^{-1}$	0.56 (-0.65 - 2.11, $5.0x10^{-1}$)	-0.20 (0.11)	$6.52x10^{-1}$	1.44 (-0.65 - 3.74, $6.8x10^{-1}$)
rs6656401	*CR1*	-0.17 (0.01)	-0.12 (0.07)	$7.04x10^{-2}$	0.73 (-0.05 - 1.58, $5.1x10^{-1}$)	-0.18 (0.10)	$8.97x10^{-1}$	1.09 (-0.32 - 2.52, $9.0x10^{-1}$)
rs200538373	*ABCA7 (G>C)*	-0.65 (0.14)	0.13 (0.51)	$7.94x10^{-1}$	-0.21 (-1.93 - 1.49, $1.4x10^{-1}$)	-0.99 (0.64)	$6.43x10^{-1}$	1.54 (-0.85 - 4.15, $6.3x10^{-1}$)
rs9331896	*CLU*	0.15 (0.01)	0.17 (0.06)	$3.73x10^{-3}$ *	1.14 (0.36 - 2.00, $7.1x10^{-1}$)	0.06 (0.08)	$3.96x10^{-1}$	0.43 (-0.92 - 1.73, $3.9x10^{-1}$)
rs35349669	*INPP5D*	0.08 (0.01)	0.09 (0.05)	$1.23x10^{-1}$	1.15 (-0.06 - 2.53)	0.04 (0.08)	$6.98x10^{-1}$	0.51 (-2.23 - 3.08, $7.1x10^{-1}$)

rs17125944	*FERMT2*	0.13 (0.02)	-0.01 (0.09)	8.75×10^{-1}	-0.11 (-1.30 - 1-07)	0.03 (0.13)	5.20×10^{-1}	0.26 (-2.21 - 2.60, 7.1×10^{-1})
rs190982	*MEF2C*	0.08 (0.01)	0.00 (0.05)	9.94×10^{-1}	0.01 (-1.25 - 1.27)	-0.06 (0.08)	1.48×10^{-1}	-0.84 (-3.81 - 1.61, 1.4×10^{-1})
	**AVERAGE**			**0.94 ± 0.12, *p* = 6.8×10^{-1}**			**1.88 ± 0.24, *p* = 1.0×10^{-4}**	

Abbreviations: *Rs ID,* variant identifier; *Gene*, gene associated with the variant; $\log OR_l^k$ *(SE),* published log(odds ratio) and relative standard error; $\log OR_{EA-NC}^k$ *(SE),* log(odds ratio) and relative standard error for comparison of extreme cases with normal controls; E_{EA-NC}^k *(95% CI, p),* increased effect-size relative to published effect-size, 95% confidence interval and p-value of difference relative to published effect sizes; $\log OR_{NA-EC}^k$ *(SE),* log(odds ratio) and relative standard error for comparison of normal AD cases with centenarian controls; E_{NA-EC}^k *(95% CI, p),* increased effect-size, 95% confidence intervals and p-value of difference relative to published effect-size; Bold type: variants with significant E_{1-2}^k; Stars*: p-value significant at nominal threshold ($p<0.05$); Asterisk$^{\#}$: p-value of association of Normal Controls (*NC*) and Extreme Controls (*EC*).

Table S4: Comparison between using younger early-onset cases (AAO <60 years) early-onset cases (AAO <65 years) and using late-onset AD cases (AAO >65 years) relative to normal controls

			Younger early-onset AD cases (AAO < 60 y.o) vs. Normal Controls (yEA vs. NC)			Early-onset cases (AAO < 65 y.o) vs. Normal Controls (eEA vs. NC)			Late-onset cases (AAO > 65 y.o) vs. Normal Controls (lEA vs. NC)		
Rs ID	***Gene***	$\log OR_l^k$ ***(SE)***	$\log OR_{yEA-NC}^k$ ***(SE)***	***P-value$_{yEA-NC}$***	E_{yEA-NC}^k **(95% CI, *p*)**	$\log OR_{eEA-NC}^k$ ***(SE)***	***P-value$_{eEA-NC}$***	E_{eEA-NC}^k **(95% CI, *p*)**	$\log OR_{lEA-NC}^k$ ***(SE)***	***P-value$_{lEA-NC}$***	E_{lEA-NC}^k **(95% CI, *p*)**
rs75932628	*TREM2 (R47H)*	0.89 (0.09)	0.48 (1.24)	6.97x10^{-1}	0.54 (-2.25 - 3.29, 7.4x10^{-1})	1.41 (0.83)	9.02x10^{-2}	1.58 (-0.25 - 3.58, 5.5x10^{-1})	1.57 (0.68)	2.16x10^{-2} *	1.78 (0.28 - 3.41, 3.2x10^{-1})
rs7920721	*ECHDC3*	0.07 (0.01)	0.16 (0.09)	9.11x10^{-2}	2.44 (-0.41 - 5.31, 3.2x10^{-1})	0.16 (0.07)	3.64x10^{-2} *	2.38 (0.12 - 5.32, 2.3x10^{-1})	0.18 (0.07)	7.66x10^{-3} *	2.70 (0.69 - 5.44, 9.9x10^{-2})
rs10498633	*SLC24A4-RIN3*	-0.09 (0.02)	-0.27 (0.12)	2.26x10^{-2}	2.88 (0.39 - 5.38, 1.4x10^{-1})	-0.16 (0.09)	8.34x10^{-2}	1.66 (-0.19 - 3.94, 5.1x10^{-1})	-0.19 (0.08)	1.96x10^{-2} *	2.01 (0.35 - 4.18, 2.5x10^{-1})
rs2718058	*NME8*	-0.08 (0.01)	0.11 (0.10)	2.68x10^{-1}	-1.40 (-3.90 - 1.08, 5.9x10^{-2})	-0.01 (0.08)	9.40x10^{-1}	0.08 (-1.95 - 2.16, 3.7x10^{-1})	-0.05 (0.07)	4.98x10^{-1}	0.61 (-1.23 - 2.53, 6.7x10^{-1})
rs72824905	*PLCG2*	-0.39 (0.06)	-0.56 (0.57)	3.28x10^{-1}	1.45 (-1.42 - 4.31, 7.5x10^{-1})	-0.38 (0.41)	3.63x10^{-1}	0.97 (-1.19 - 3.24, 9.7x10^{-1})	-0.54 (0.39)	1.63x10^{-1}	1.40 (-0.61 - 3.69, 7.0x10^{-1})
rs9271192	*HLA-DRB1*	-0.11 (0.01)	-0.07 (0.10)	5.22x10^{-1}	0.61 (-1.28 - 2.54, 6.8x10^{-1})	-0.21 (0.12)	6.50x10^{-2}	1.98 (-0.11 - 4.43, 3.6x10^{-1})	-0.07 (0.11)	5.11x10^{-1}	0.65 (-1.34 - 2.72, 7.1x10^{-1})
**rs1476679**	***ZCWPW1***	**0.09 (0.01)**	-0.01 (0.10)	8.79x10^{-1}	-0.20 (-2.43 - 1.99, 2.8x10^{-1})	**0.01 (0.08)**	**9.13x10^{-1}**	**0.10 (-1.73 - 1.98, 3.1x10^{-1})**	**0.24 (0.07)**	**1.15x10^{-3} ***	**2.73 (1.04 - 4.96, 4.3x10^{-2})**
rs4147929	*ABCA7 (A>G)*	-0.14 (0.02)	-0.27 (0.12)	2.50x10^{-2}	1.88 (0.23 - 3.51, 2.9x10^{-1})	-0.19 (0.10)	5.35x10^{-2}	1.30 (-0.01 - 2.81, 6.4x10^{-1})	-0.16 (0.09)	7.30x10^{-2}	1.10 (-0.12 - 2.43, 8.8x10^{-1})
**rs7412**	***APOE (ε2)***	**-0.79 (0.03)**	-1.61 (0.32)	7.26x10^{-7}	2.05 (1.22 - 2.87, 1.3x10^{-2})	**-1.41 (0.22)**	**3.77x10^{-10} ***	**1.80 (1.21 - 2.40, 8.6x10^{-3})**	**-0.89 (0.16)**	**2.52x10^{-8} ***	**1.13 (0.72 - 1.56, 5.1x10^{-1})**
rs13113697	*HS3ST1*	-0.07 (0.01)	0.13 (0.11)	2.41x10^{-1}	-1.93 (-5.15 - 1.28, 7.5x10^{-2})	0.03 (0.08)	7.16x10^{-1}	-0.46 (-3.29 - 2.12, 2.5x10^{-1})	-0.03 (0.08)	7.37x10^{-1}	0.38 (-1.94 - 2.86, 5.9x10^{-1})
rs616338	*ABI3*	-0.36 (0.05)	-0.75 (0.42)	7.37x10^{-2}	2.06 (-0.19 - 4.39, 3.6x10^{-1})	-0.49 (0.37)	1.90x10^{-1}	1.35 (-0.68 - 3.60, 7.4x10^{-1})	-0.82 (0.31)	8.01x10^{-3} *	2.27 (0.61 - 4.43, 1.3x10^{-1})
rs10948363	*CD2AP*	0.10 (0.01)	-0.00 (0.11)	9.79x10^{-1}	-0.01 (-2.20 - 2.18, 3.7x10^{-1})	0.06 (0.08)	4.89x10^{-1}	0.60 (-1.11 - 2.47, 6.4x10^{-1})	0.07 (0.07)	3.23x10^{-1}	0.77 (-0.77 - 2.45, 7.7x10^{-1})
rs429358	*APOE (ε4)*	1.05 (0.03)	1.27 (0.11)	8.69x10^{-31}	1.21 (1.01 - 1.42, 3.6x10^{-2})	1.32 (0.09)	3.76x10^{-50} *	**1.26 (1.08 - 1.44, 3.4x10^{-3})**	1.42 (0.08)	1.86x10^{-66} *	1.36 (1.19 - 1.53, 4.5x10^{-4})
rs11771145	*EPHA1*	-0.10 (0.01)	-0.04 (0.10)	7.06x10^{-1}	0.37 (-1.61 - 2.29, 5.1x10^{-1})	0.00 (0.08)	9.76x10^{-1}	-0.02 (-1.55 - 1.57, 2.1x10^{-1})	-0.17 (0.07)	1.63x10^{-2} *	1.70 (0.32 - 3.36, 3.2x10^{-1})
rs10838725	*CELF1*	0.08 (0.01)	0.25 (0.10)	1.17x10^{-2}	3.15 (0.70 - 5.60, 8.5x10^{-2})	0.13 (0.08)	9.61x10^{-2}	1.66 (-0.30 - 4.04, 5.1x10^{-1})	0.02 (0.07)	7.94x10^{-1}	0.24 (-1.65 - 2.17, 4.2x10^{-1})
rs28834970	*PTK2B*	0.10 (0.01)	0.25 (0.09)	9.04x10^{-3}	2.50 (0.60 - 4.42, 1.2x10^{-1})	0.12 (0.07)	1.06x10^{-1}	1.23 (-0.27 - 2.92, 7.7x10^{-1})	0.05 (0.07)	4.59x10^{-1}	0.52 (-0.87 - 2.01, 5.1x10^{-1})
**rs983392**	***MS4A6A***	-0.11 (0.01)	0.09 (0.09)	3.20x10^{-1}	-0.87 (-2.62 - 0.89, 6.8x10^{-2})	**0.12 (0.07)**	**1.16x10^{-1}**	**-1.11 (2.62 - 0.24, 2.8x10^{-3})**	**-0.13 (0.07)**	**6.31x10^{-2}**	1.19 (-0.04 - 2.63, 7.6x10^{-1})
rs11218343	*SORL1*	-0.26 (0.03)	0.04 (0.24)	8.73x10^{-1}	-0.13 (-1.92 - 1.66, 2.0x10^{-1})	-0.14 (0.20)	4.78x10^{-1}	0.54 (-0.92 - 2.11, 5.4x10^{-1})	-0.27 (0.19)	1.54x10^{-1}	1.02 (-0.39 - 2.55, 9.8x10^{-1})
rs6733839	*BIN1*	0.20 (0.01)	0.21 (0.09)	2.49x10^{-2}	1.07 (0.12 - 2.01, 8.7x10^{-1})	0.19 (0.07)	1.16x10^{-2} *	0.95 (0.23 - 1.73, 9.1x10^{-1})	0.15 (0.07)	2.36x10^{-2} *	0.78 (0.10 - 1.48, 5.2x10^{-1})
rs10792832	*PICALM*	0.14 (0.01)	0.16 (0.10)	1.13x10^{-1}	1.11 (-0.30 - 2.51, 8.8x10^{-1})	0.18 (0.08)	2.10x10^{-2} *	1.28 (0.20 - 2.43, 6.1x10^{-1})	0.16 (0.07)	2.33x10^{-2} *	1.12 (0.16 - 2.18, 8.1x10^{-1})
rs7274581	*CASS4*	-0.13 (0.02)	-0.26 (0.18)	1.54x10^{-1}	1.98 (-0.75 - 4.71, 4.8x10^{-1})	-0.34 (0.15)	2.31x10^{-2} *	2.53 (0.39 - 5.43, 1.6x10^{-1})	-0.04 (0.12)	7.18x10^{-1}	0.33 (-1.60 - 2.24, 4.6x10^{-1})
rs143332484	*TREM2 (R62H)*	0.50 (0.07)	0.17 (0.37)	6.52x10^{-1}	0.34 (-1.10 - 1.80, 3.7x10^{-1})	0.25 (0.28)	3.73x10^{-1}	0.51 (-0.62 - 1.67, 3.9x10^{-1})	-0.03 (0.28)	9.19x10^{-1}	-0.06 (-1.22 - 1.09, 6.9x10^{-2})
rs118172952	*KANSL1*	-0.14 (0.03)	-0.17 (0.14)	2.22x10^{-1}	1.25 (-0.74 - 3.27, 8.0x10^{-1})	0.01 (0.10)	8.95x10^{-1}	-0.10 (-1.86 - 1.57, 1.8x10^{-1})	-0.15 (0.10)	1.26x10^{-1}	1.09 (-0.33 - 3.20, 9.1x10^{-1})
rs6656401	*CR1*	-0.17 (0.01)	-0.31 (0.11)	4.39x10^{-3}	1.87 (0.58 - 3.15, 1.9x10^{-1})	-0.17 (0.09)	5.43x10^{-2}	1.02 (0.01 - 2.14, 9.6x10^{-1})	-0.08 (0.08)	3.04x10^{-1}	0.50 (-0.46 - 1.49, 3.0x10^{-1})
rs200538373	*ABCA7 (G>C)*	-0.65 (0.14)	0.00 (0.90)	9.97x10^{-1}	-0.02 (-2.79 - 2.74, 4.7x10^{-1})	0.29 (0.62)	6.39x10^{-1}	-0.45 (-2.63 - 1.52, 1.3x10^{-1})	-0.03 (0.64)	9.57x10^{-1}	0.05 (-2.10 - 2.21, 3.6x10^{-1})
rs9331896	*CLU*	0.15 (0.01)	0.28 (0.10)	5.39x10^{-3}	1.92 (0.59 - 3.23, 1.8x10^{-1})	0.13 (0.08)	1.03x10^{-1}	0.86 (-0.16 - 1.93, 7.9x10^{-1})	0.20 (0.07)	4.89x10^{-3} *	1.34 (0.41 - 2.37, 4.6x10^{-1})
rs35349669	*INPP5D*	0.08 (0.01)	0.14 (0.10)	1.52x10^{-1}	1.82 (-0.68 - 4.35, 5.2x10^{-1})	0.15 (0.07)	4.23x10^{-2} *	2.04 (0.04 - 4.59, 3.1x10^{-1})	0.04 (0.07)	5.59x10^{-1}	0.52 (-1.33 - 2.43, 6.0x10^{-1})

rs17125944	*FERMT2*	0.13 (0.02)	-0.15 (0.16)	3.44×10^{-1}	-1.17 (-3.63 - 1.24, 7.7×10^{-2})	0.01 (0.12)	9.25×10^{-1}	0.08 (-1.83 - 2.01, 3.2×10^{-1})	-0.04 (0.11)	7.47×10^{-1}	-0.27 (-2.12 - 1.45, 1.3×10^{-1})
rs190982	*MEF2C*	0.08 (0.01)	-0.11 (0.09)	2.52×10^{-1}	-1.43 (-3.91 - 1.03, 5.3×10^{-2})	-0.05 (0.07)	4.98×10^{-1}	-0.67 (-2.85 - 1.33, 9.7×10^{-2})	0.03 (0.07)	6.12×10^{-1}	0.45 (-1.32 - 2.41, 5.6×10^{-1})
**AVERAGE**			**0.87 ± 0.20, p = 7.3×10^{-1}**			**0.86 ± 0.16, *p* = 7.9×10^{-1}**			**1.01 ± 0.14, *p* = 4.6×10^{-1}**		

Abbreviations: *Rs ID*, variant ID; *Gene*, gene associated with the variant according to paper in which was first associated; $\log OR_l^k$, published log(odds ratio) and relative standard error, *i.e* the first or largest case-control study that reported the variant association with AD; $\log OR_{yEA-NC}^k$, Effect-size and relative standard error of AD association of 255 young AD cases (age at onset <60 years of age) *vs.* 1,664 normal controls; E_{yEA-NC}^k(95% CI, *p*), change in effect size when using young AD cases (age at onset < 60 years of age), 95% confidence intervals and p-value of difference relative to published effect sizes; $\log OR_{eEA-NC}^k$, Effect-size and relative standard error of AD association of 464 early-onset AD cases *vs.* normal controls; E_{eEA-NC}^k(95% CI, *p*), increased effect size when using extreme AD early-onset, 95% confidence intervals and p-value of difference relative to published effect sizes; $\log OR_{lEA-NC}^k$, Effect-size and relative standard error of association of 609 late-onset AD cases *vs.* normal controls; E_{lEA-NC}^k (95% CI, *p*), increased effect size when using extreme AD late-onset, 95% confidence intervals and p-value of difference relative to published effect sizes; Bold type: variants with a significant difference between the effect-sizes of 464 early-onset cases (age at onset <65) and 609 late-onset cases (age at onset >65) relative to 1,664 normal controls; Stars*: p-value significant at nominal threshold (*p*<0.05).

Table S5: Functional implication of all the tested variants

Locus	*Gene*	*Variant*	*GO*	*Literature*	E^{k}_{EA-EC}
6:41129252	*TREM2 R47H*	rs75932628	positive regulation of antigen processing and presentation of peptide antigen via MHC class II (GO:0002588); phagocytosis (GO:0006911); humoral immune response (GO:0006569)	-	6.46
10:11720308	*ECHDC3*	rs7920721	metabolic process (GO:0008152)	-	4.56
14:92926952	*SLC24A4*	rs10498633	transport (GO:0006810); ion transport (GO:0006811); sodium ion transport (GO:0006814)	Neural development and regulation of blood pressure and hypertension	4.50
7:37841534	*NME8*	rs2718058	nucleoside diphosphate phosphorylation (GO:0006165); multicellular organism development (GO:0007275); cell differentiation (GO:0030154)	Cytoskeletal function and axonal transport	3.80
16:81942028	*PLCG2*	rs72824905	positive regulation of receptor internalization (GO:0002092); follicular B cell differentiation (GO:0002316); lipid metabolic process (GO:0006629)	-	3.28
6:32578530	*HLA-DRB1*	rs111418223	immune system process (GO:0002376); immunoglobulin production involved in immunoglobulin mediated immune response (GO:0002381); inflammatory response to antigenic stimulus (GO:0002437)	Immune response	3.20
7:100004446	*ZCWPW1*	rs1476679	-	Epistatis regulation and Neural development	2.97
19:1063443	*ABCA7 A>G*	rs4147929	transport (GO:0006810); phagocytosis (GO:0006909); positive regulation of cholesterol efflux (GO:0010875)	Lipid metabolism and Immune system	2.26
19:45412079	*APOE ε2*	rs7412	response to reactive oxygen species (GO:0000302); retinoid metabolic process (GO:0001523); negative regulation of endothelial cell proliferation (GO:0001937)	Lipid metabolism and cholesterol transport	2.24
17:47297297	*ABI3*	rs616338	movement of cell or subcellular component (GO:0006928); regulation of cell migration (GO:0030334)	-	2.06
4:11711232	*HS3ST1*	rs13113697	glycosaminoglycan biosynthetic process (GO:0006024)	-	2.06
6:47487762	*CD2AP*	rs10948363	protein complex assembly (GO:0006461); substrate-dependent cell migration, cell extension (GO:0006930); cell cycle (GO:0007049)	Endosomal vesicle cycling	2.00

19:45411941	*APOE ε4*	rs429358	response to reactive oxygen species (GO:0000302); retinoid metabolic process (GO:0001523); negative regulation of endothelial cell proliferation (GO:0001937)	Lipid metabolism and cholesterol transport	1.99
7:143110762	*EPHA1*	rs11771145	angiogenesis (GO:0001525); positive regulation of cell-matrix adhesion (GO:0001954); protein phosphorylation (GO:0006468)	Endosomal vescicle cycling and Immune system	1.94
11:47557871	*CELF1*	rs10838725	mRNA processing (GO:0006376); germ cell development (GO:0007281); mRNA splice site selection (GO:0006376)	Cytoskeletal function and axonal transport	1.78
8:27195121	*PTK2B*	rs28834970	MAPK cascade (GO:0000165); response to reactive oxygen species (GO:0000302); angiogenesis (GO:0001525)	Cell migration and synaptic function	1.76
11:59923508	*MS4A6A*	rs983392	-	Immune response	1.56
11:121435587	*SORL1*	rs11218343	protein targeting (GO:0006605); lipid metabolic process (GO:0006629); transport (GO:0006810)	Endosomal vesicle cycling	1.48
2:127892810	*BIN1*	rs6733839	endocytosis (GO:0006897); nucleus organization (GO:0006997); multicellular organism development (GO:0007275)	Endosomal vesicle cycling	1.28
11:85867875	*PICALM*	rs10792832	protein complex assembly (GO:0006461); endocytosis (GO:0006897); axonogenesis (GO:0007409)	Endosomal vesicle cycling	1.09
20:55018260	*CASS4*	rs7274581	cell adhesion (GO:0007155)	Cytoskeletal function and axonal transport	1.06
17:44353222	*KANSL1*	rs118172952	covalent chromatin modification (GO:0016569); histone H4-K5 acetylation (GO:0043981); histone H4-K8 acetylation (GO:0043982)	-	0.97
6:41129207	*TREM2 R62H*	rs143332484	positive regulation of antigen processing and presentation of peptide antigen via MHC class II (GO:0002588); phagocytosis (GO:0006911); humoral immune response (GO:0006569)	-	0.97
1:207692049	*CR1*	rs6656401	immune system process (GO:0002376); complement receptor mediated signaling pathway (GO:0002430); complement activation (GO:0006958)	Immune response	0.75
19:1061892	*ABCA7 G>C*	rs200538373	transport (GO:0006810); phagocytosis (GO:0006909); positive regulation of cholesterol efflux (GO:0010875)	Lipid metabolism and Immune system	0.68
8:27467686	*CLU*	rs9331896	cell morphogenesis (GO:0000902); microglial cell activation (GO:0001774); immune system process (GO:0002376)	Cholesterol and Lipid metabolism and Immune system	0.60
2:234068476	*INPP5D*	rs35349669	phosphatidylinositol biosynthetic process (GO:0006661); phosphate-containing compound metabolic process (GO:0006796); apoptotic process (GO:0006915)	Immune response	0.36

14:53400629	*FERMT2*	rs17125944	cell adhesion (GO:0007155); cell-matrix adhesion (GO:0007160); regulation of cell shape (GO:0008360)	Cytoskeletal function and axonal transport	-0.82
5:88223420	*MEF2C*	rs190982	negative regulation of transcription by RNA polymerase II (GO:0000122); MAPK cascade (GO:0000165); blood vessel development (GO:0001568)	Immune response, Neural development and synaptic functions	-1.86

Abbreviations: Locus, genomic location of the variant; Gene, the nearest gene associated with the variant in the paper that first associated the variant with AD; RsID, variant identifier, GO annotation, first three Gene Ontology (GO) biological processes associated with gene; Literature annotation, pathway(s) associated with each gene according to a recent review regarding the genetic landscape of Alzheimer's disease[43]; E^{k}_{EA-EC}, effect-size change when using extreme phenotypes relative to published effect size. Orange cells indicate variants with a decreased effect-size as compared to published effect-size. Green cells correspond to variants with an increased effect-size as compared to published effect-size.

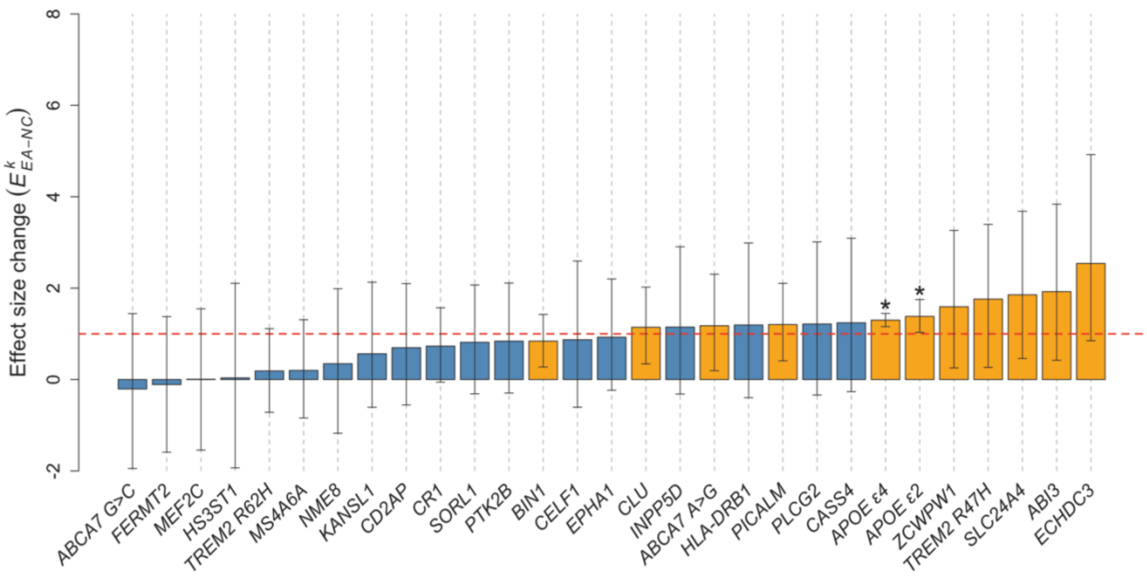

***Figure S1*: Extreme AD cases vs. normal controls: $\boldsymbol{E^{k}_{EA-NC}}$.** The effect-size change was significant for 4 variants (*p*<0.05, two-sample z-test; bars annotated with a star [*]). Orange bars indicate nominal statistical significance for the association with AD (*p*<0.05). Dashed red line (E^{k}_{EA-NC} =1) indicates same effect-size as reported in literature.

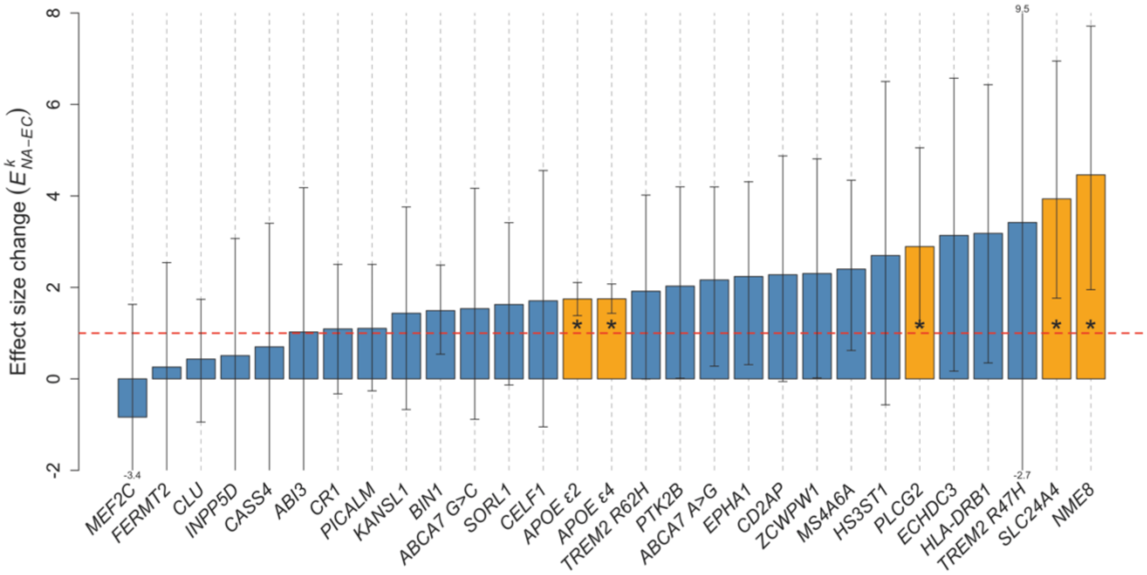

***Figure S2*: Normal ADs vs. Extreme (centenarian) controls: $\boldsymbol{E^{k}_{NA-EC}}$.** Effect-size change ($E^{k}_{NA-EC}$) was significant for 5 variants (*p*<0.05, two-sample z-test; bars annotated with a star [*]). Orange bars indicate nominal statistical significance for the association with AD (*p*<0.05). Dashed red line indicates same effect-size as reported in literature.

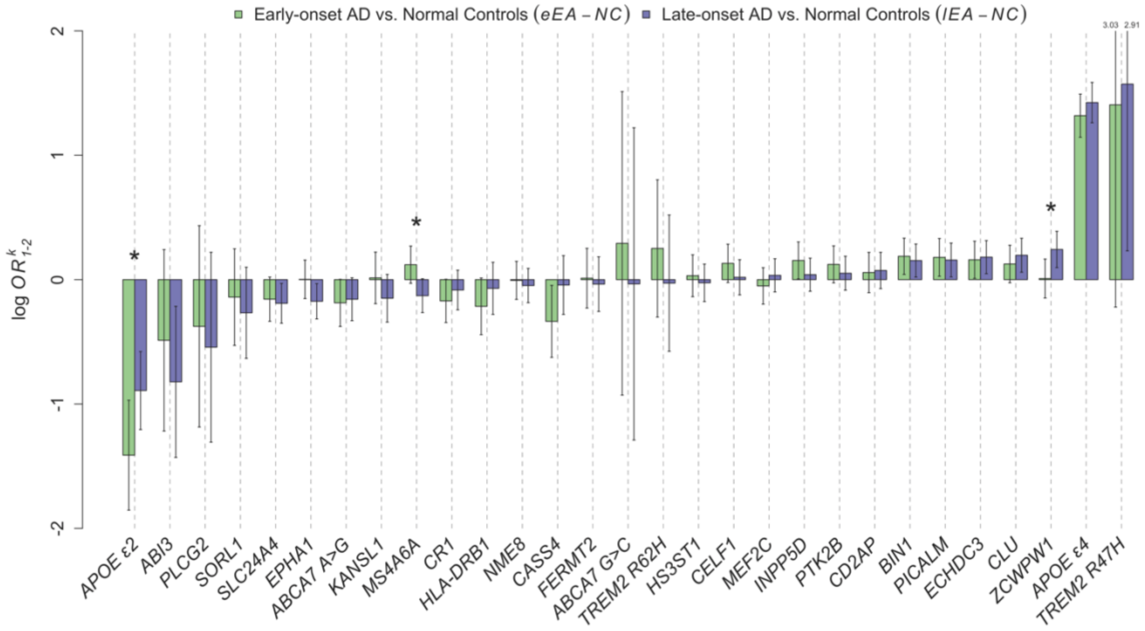

Figure S3: **Early onset AD vs normal controls and late onset AD vs normal controls.** Effect-sizes and 95% confidence intervals of a comparison of early onset AD cases ($\log OR^{k}_{eEA-NC}$, age at onset ≤ 65 years) and late-onset AD ($\log OR^{k}_{lEA-NC}$, age at onset > 65 years) with normal controls. For all the variants, the 95% confidence intervals overlapped. [*]: difference between $\log OR^{k}_{eEA-NC}$ and $\log OR^{k}_{lEA-NC}$ was significant (p<0.05, two-sample z-test).

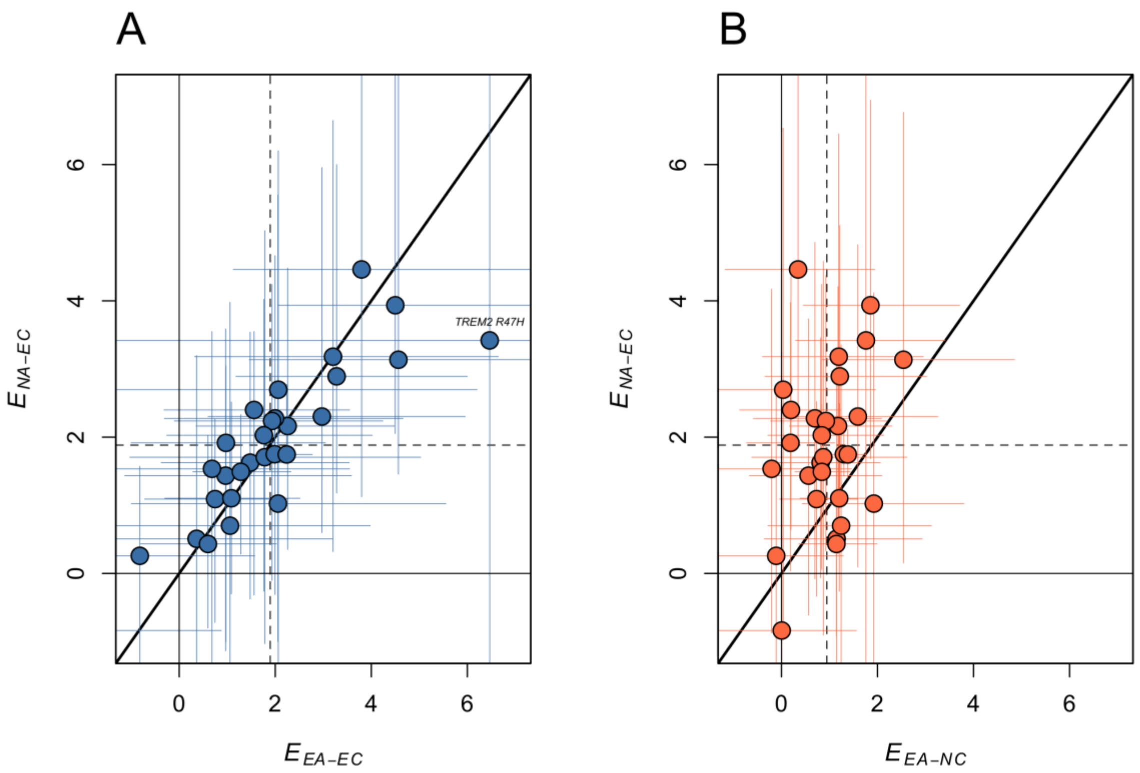

Figure S4: **Comparison of effect size changes at the variant level**

A: Effect of using extreme AD cases vs normal AD cases: X-axis: $E^{k}_{EA\text{-}EC}$: Effect size changes from a comparison of the extreme cases and extreme (centenarian) controls relative to published effect sizes. Dashed line x-axis average effect-size increase $E^{k}_{EA\text{-}EC}$ at 1.90 ±0.29: Y-axis: $E^{k}_{NA\text{-}EC}$: effect size changes from a comparison normal AD cases with extreme (centenarian) controls relative to published effect sizes. Dashed line y-axis: average effect-size increase $E^{k}_{NA\text{-}EC}$ at 1.88 ± 0.24. See *Table 2* for $E^{k}_{EA\text{-}EC}$ and *Table S3* for $E^{k}_{NA\text{-}EC}$ values **B. Effect of using extreme cases vs using extreme controls:** X-axis: effect-size changes of extreme AD cases *vs*. normal controls relative to published effect-sizes. Dashed line x-axis: average effect-size increase $E^{k}_{EA\text{-}NC}$ at 0.94 ± 0.12. Y-axis: Variant effect-size change of normal AD cases *vs*. extreme controls relative to published effect-sizes. Dashed line y-axis: average effect-size increase $E^{k}_{NA\text{-}EC}$ at 1.88 ± 0.24. See *Table S3* for $E^{k}_{EA\text{-}NC}$ and $E^{k}_{NA\text{-}EC}$ values.
